# Development of Bifunctional Chiral Thioureas and Thiosquaramides in the Synthesis of Betti Bases

**DOI:** 10.3390/molecules28237835

**Published:** 2023-11-29

**Authors:** Martyna Malinowska, Anna Zawisza

**Affiliations:** Department of Organic and Applied Chemistry, Faculty of Chemistry, University of Lodz, Tamka 12, 91-403 Lodz, Poland; martyna.malinowska@chemia.uni.lodz.pl

**Keywords:** asymmetric organocatalysis, bifunctional organocatalyst, thiourea, thiosquaramide, Betti bases

## Abstract

Bifunctional thioureas and, for the first time, bifunctional thiosquaramides as organocatalysts were used in the asymmetric Betti reaction involving 1-, 2-naphthols and hydroxyquinoline with *N*-tosylimine and ketimine. The described methodology affords direct access to chiral aminoarylnaphthols in excellent yield (up to 98%) with high enantioselectivity (up to 80% ee) and enantioenriched 3-amino-2-oxindoles (up to 78% yield, up to 98% ee).

## 1. Introduction

The Betti reaction, alternatively referred to as the Aza-Friedel–Crafts reaction, represents an important variant of the Mannich reaction and is one of the most important C-C bond-forming reactions in organic chemistry. The Betti reaction is a simple multicomponent condensation between 2-naphthol, aryl aldehydes, and amines that leads to aminobenzylnaphthols, the so-called Betti bases [1,2,3]. Over the past decade, many research groups have reported different variants of this reaction, generally using a pre-formed imine that is often protected as a sulfonamide [4,5,6]. Aminoarylnaphthols are a class of molecules found in natural and synthetic compounds with a wide range of interesting activities and applications. Chiral Betti bases have been widely used in optoelectronics, but their biological properties, such as anticancer, anti-bacterial, antioxidant, anti-inflammatory, antipain, antihypertensive, anti-Alzheimer, and bradycardia activities, deserve special attention [7]. In addition, chiral Betti bases have been proven to be useful ligands and auxiliaries in asymmetric synthesis [7,8,9,10]. Their widespread applications have generated considerable interest in developing asymmetric methods for their preparation. In 2010, the Hui group reported an enantioselective Betti reaction of 2-naphthol with tosylimines catalyzed by a dinuclear zinc complex, to prepare chiral Betti base derivatives with high yields and excellent enantioselectivity of up to 96% [11]. Subsequent studies were carried out using quinine-squaramide organocatalysts [12]. The results obtained by Chimni and Wang’s group demonstrated that α- and β-naphthol derivatives react readily with various aromatic and heteroaromatic *N*-sulfonylimines in the presence of *Cinchona* alkaloid derived bifunctional organocatalyst [13,14]. The reaction of naphthols with isatin-derived ketimines turned out to be more demanding. In 2015, Pedro and Khan’s groups, respectively, reported an enantioselective version of this reaction organocatalyzed by the same quinine-derived thiourea to yield chiral 3-amino-2-oxindoles in high yields with excellent enantioselectivity [15,16,17]. Two years later, Tanyeli used bulky cinchona-derived squaramides in the same transformation on naphthols [18]. In 2020, Pedrosa and coworkers reported that aminoethyl polystyrene-supported cinchona-thiourea derivatives could be recovered and reused in a highly enantioselective aza-Friedel–Crafts reaction of different naphthols with a variety of *N*-Boc ketimines derived from isatin [19]. Recently, Wang and Jin reported simple Takemoto-type thiourea-catalyzed reactions of β-naphthols with isatines leading to 3-(naphthalen-1-yl)-3-amino-2-oxindoles with excellent 89–95% yield and high enantioselectivity (90–97% ee) [20]. We would also like to mention the enantio- and diastereoselective cascade Betti/aza-Michael reaction of phenols and *N*-tosylimin containing Michael acceptor catalyzed by bifunctional thiourea organocatalysts leading to 1,3-disubstituted isoindolines [21]. To our knowledge, these are the most important, if not the only, literature reports on the asymmetric Betti reaction. Due to the high demand for the development of this reaction, we decided to test thiourea and, for the first time, thiosquaramide catalysts, compare the obtained results with those already published, and then draw general conclusions regarding the use of organocatalysts of this type in the reactions of α- and β-naphthols and 6-hydroxyquinoline with tosylimine and ketimine. Catalysts of this type have a number of advantages: their synthesis is uncomplicated, and the reactions involving them proceed under mild conditions, without metal cation. Moreover, the presence of a thiourea/thiosquaramide bridge and strongly acidic protons leads to the formation of strong hydrogen bonds and activation of the substrate. While bifunctional chiral thioureas have been widely used in asymmetric synthesis [22,23,24], including the Betti reaction, bifunctional chiral thiosquaramides were used only in Michael addition [25,26,27,28,29], asymmetric Michael–Henry tandem reaction [30], and the addition of lawsone to β,γ-unsaturated α-keto ester [31].

## 2. Results and Discussion

**Figure 1:**
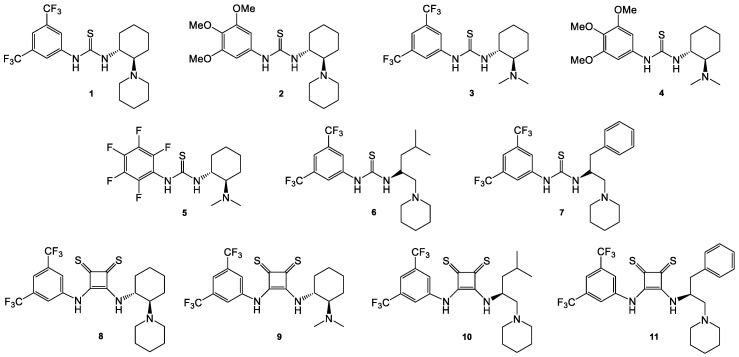
Thiourea and thiosquaramide derivatives screened as organocatalysts.

Bifunctional thioureas were prepared in one step, as previously described [32,33,34]. The reaction of appropriate, commercially available isothiocyanates with amines leads to thioureas in near quantitative yields. Two of the synthesized thioureas, **2** and **5**, are new compounds, while the bifunctional Takemoto catalyst **3** is commercially available (Figure 1). Thiosquaramides **8**–**11** were prepared in four steps from 3,4-dihydroxycyclobut-3-en-1,2-dione (squaric acid). In the first step, esterification of the square acid with cyclopentanol gave dicyclopentyl squarate [25,31]. Subsequently, dithionation with Lawesson’s reagent led to dicyclopentyl dithiosquarate, which was found to be an excellent platform for the synthesis of **8**–**11** (Figure 1). Disubstituted thiosquaramide derivatives reacted vigorously with primary amines—first with 3,4-bis(trifluoromethyl)aniline and then with the appropriate amine having a chiral center and a tertiary amine. The last step was carried out using the traditional solvent method, but also using mechanochemistry. It should be emphasized that the use of a ball mill resulted in a significant increase in chemical efficiency. Due to the possibility of creating rotamers and zwitterionic species by bifunctional thiosquaramides, especially those possessing an aryl substituent, we transformed them into appropriate hydrochlorides, which resulted in simplifying their spectra and sharpening many signals [31].

**Scheme 1:**
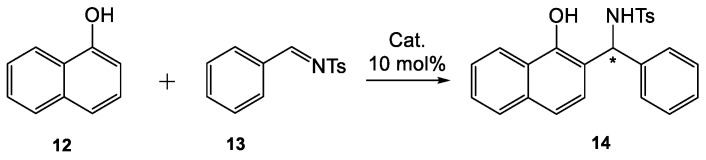
Betti reaction of 1-naphthol (**12**) with *N*-tosylimine **13**.

To study the feasibility and enantioselectivity of the Betti reaction, a bifunctional catalyst **1** which consists of H-bond donor and amine moieties was screened for a model reaction of 1-naphthol (**12**) with *N*-tosylimine (**13**) in toluene at room temperature (Scheme 1 and Table 1). The desired product **14** was isolated in good yield (98%) and with 75% ee after 20 h (Table 1, entry 2) when a threefold excess of 1-naphthol relative to imine was used.

To further improve the enantioselectivity of the transformation, we investigated a variety of different reaction conditions, including the solvent, temperature, and catalyst loading (Table 2).

The obtained results revealed that the solvent effect had a significant impact on the efficiency of the reaction. Toluene was found to be optimal (Table 1, entry 2), while reactions carried out in THF, ACN, *o*-xylene, and DCM resulted in a decrease in yield and enantioselectivity (Table 1, entries 9–12). The screening of catalyst loading exhibited that 10 mol% equivalent of **1** was optimal, while 5 mol% and 30 mol% offered no improvement in the asymmetric induction (Table 1, entries 7–8). It is noteworthy that the temperature also influenced the reaction—after lowering it from rt to 0 °C, a slight increase in enantioselectivity was observed (Table 1, entry 6). Based on these experiments, the optimized conditions were determined to be toluene as the solvent with a 10 mol% loading of catalyst **1** at 0 °C (Table 1, entry 6). The absolute configuration of the product was assigned as (*S*) based on a comparison of the optical rotation of the product material with a value from the literature [14,32].

With the optimized conditions in hand, we evaluated the general applicability of this asymmetric Betti reaction using bifunctional thioureas **1**–**7** (Table 2 entries 1–7). All tested organocatalysts gave the expected product in high yields (80–98%) with moderate to high enantioselectivities (58–80% ee). (*R*,*R*)-piperidine-based thiourea **1** and Takemoto catalyst **3** with the 3,5-bis(trifluoromethyl)phenyl moiety provided the best yield and enantioselectivity (Table 2, entries 1 and 3). The obtained results prove the higher efficiency of thiourea catalysts having at one of the nitrogen atoms an aryl group with strongly electron-acceptor substituents (CF_3_), which by increasing the acidity of the N-H bond facilitates the formation of a hydrogen bond with the substrate. In contrast, bifunctional organocatalysts **6**–**7** without the aminocyclohexyl moiety showed lower asymmetric induction (Table 2, entries 6–7).

Thiosquaramide organocatalysts **8**–**11**, used for the first time in the Betti reaction, promoted the reaction but gave lower yields and ee values (Table 2, entries 8–15). Organocatalysts **8**–**9** containing ((1*R*,2*R*)-2-(dimethylamino)cyclohexyl)amine or ((1*R*,2*R*)-2-(piperidin-1-yl)cyclohexyl)amine moiety gave predominantly (*S*)-product, while those not containing these moieties had the opposite enantiomer of **14**. A similar relationship was observed for thioureas **1**–**7**. The higher activity of thiosquaramides **8**–**11** (Table 2, entries 8, 10, 12, and 14) should also be noted compared to their appropriate hydrochlorides (Table 2, entries 9, 11, 13, and 15).

**Scheme 2:**
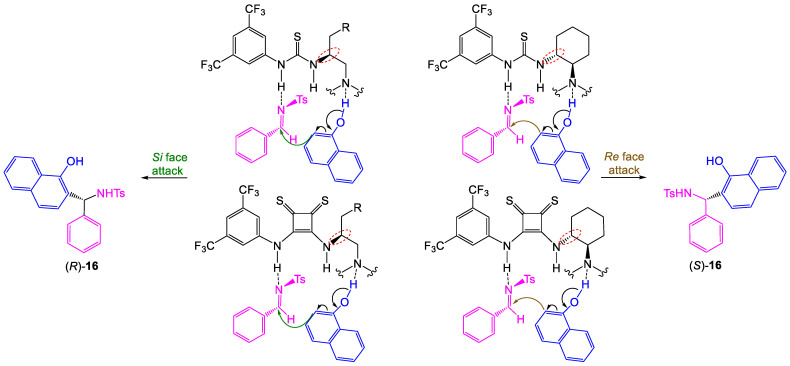
Proposed dual activation for the thiourea and thiosquaramide-catalyzed asymmetric Betti reaction of 1-naphthol with *N*-tosylimine.

To rationalize the stereochemical outcome, a dual activation for the thiourea and thiosquaramide-catalyzed asymmetric Betti reaction of 1-naphthol (**12**) with *N*-tosylimine **13** is proposed in Scheme 2. Based on previous literature reports [13,22,23,24] and the observed stereochemistry, a transition state involving a ternary complex between the catalyst and the substrates can be proposed. Both thiourea and thiosquaramide catalysts promote the reaction in a dual manner: activating *N*-tosylimine through forming H-bond with the thiourea/thiosquaramide motif and enhancing the nucleophilicity of 1-naphthol by the tertiary amine moiety of catalyst. As shown in Scheme 2, a configuration of the product was determined by the absolute configuration of the carbon atom at the thioamide nitrogen atom of the thiourea/thiosquaramide moiety. For organocatalysts **1**–**5** and **8**–**9**, the activated 1-naphthol is capable of nucleophilic attack from C2 on the *Re* face of the imine providing the *S* enantiomer of the product. On the other hand, for organocatalysts **6**–**7** and **10**–**11** with the opposite absolute configuration (the compatibility of the relative configuration results from the different priority of the substituents), the attack of the nucleophile on the *Si* face of the imine is favored which leads to (*R*)-**16**.

**Scheme 3:**
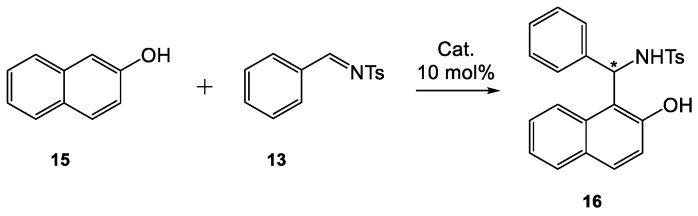
Betti reaction of 2-naphthol (**15**) with *N*-tosylimine **13**.

To demonstrate the generality of the **1**–**11**-promoted asymmetric Betti reaction, other imines and naphthols were explored (Table 3, Table 4, Table 5, Table 6 and Table 7). The optimization of the reaction conditions of 2-naphthol (**15**) with *N*-tosylimine **13** (Scheme 3) catalyzed by thiourea **1** indicated that the use of substrates in the molar ratio naphthol:imine = 3:1, 10 mol% of **1** in toluene at 0 °C was optimal for this transformation and led to chiral product **16** in 98% yield and 54% ee value (Table 3, entry 2).

In the next step, we decided to react 2-naphthol (**15**) with imine **13** in the presence of other organocatalysts **2**–**11** (Table 4). 

All tested bifunctional thioureas **1**–**7** gave product **16** in high yields (65–98%) but with low enantiomeric excesses (6–61% ee, Table 4, entries 1–7). The obtained results showed that Takemoto-type organocatalysts **1**–**5** were less effective in this transformation. In contrast, all bifunctional thiosquaramides **8**–**11** and their hydrochlorides slightly improved the asymmetric induction (Table 4, entries 8–15). Although the yields of reactions catalyzed by thiosquaramides were low compared to thioureas, the ee values were higher (46–71% ee). Nevertheless, this is the first example of the use of thiosquaramide organocatalysts in the reaction of 2-naphthol with imine **13**. Their higher catalytic activity in the reaction of 2-naphthol compared to 1-naphthol should also be noted (Table 2 vs. Table 4). 

In the next stage of our research, we decided to test the catalytic activity of thiourea **1** in the reaction of 6-hydroxyquinoline as a nucleophile (**17**) with *N*-tosylimine **13**. Unfortunately, despite many attempts, we were unable to obtain the desired product.

**Scheme 4:**
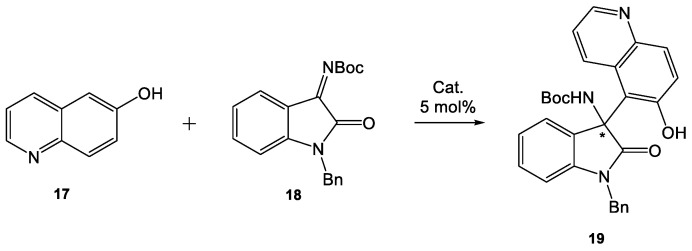
Betti reaction of 6-hydroxyquinoline (**17**) and ketimine **18**.

Based on literature reports [17], we decided to react 6-hydroxyquinoline (**17**) with isatin-derived *N*-Boc ketimine **18** (Scheme 4, Table 5). We carried out the first test under the conditions developed by Pedro for this transformation [17]. Thiourea **1** (5 mol%) catalyzed the reaction in toluene to afford regioselectively alkylated at C-5 carbon atom chiral product **19** with 68% yield after 48 h (Table 5, entry 1) with a good enantiomeric excess (76% ee). When the reaction temperature was lowered to 0 °C, the ee value could be further enhanced to 87% (Table 5, entry 2). Changing the solvent to DCM resulted in a drastic decrease in the yield and selectivity of the reaction (Table 5, entry 3). The screening of catalyst loading exhibited that 5 mol% equivalent of **1** was optimal, while 10 mol% and 30 mol% offered no improvement in the asymmetric induction (Table 5, entries 4–5).

Next, we investigated the Betti reaction under optimal conditions with the other catalysts (Table 6). Thiourea **3** proved to be the most effective in terms of enantioselectivity of all Takemoto’s type organocatalysts (92% ee), although with unsatisfactory yield—only 65% (Table 6, entries 1–6). Thioureas **6** and **7**, which lacked the cyclohexylamine grouping, performed better under these conditions (Table 6, entries 7–9) (Table 6, entries 7–9). The chiral product **19** was obtained with 75% yield and 98% ee at 0 °C. Thiosquaramides **8**–**11**, as well as their hydrochlorides, showed very low catalytic activity (Table 6, entries 10–17), although quinine-derived squaramides gave excellent enantioselectivity under these conditions, as reported by Pedro [17] and Tanyeli [18].

**Scheme 5:**
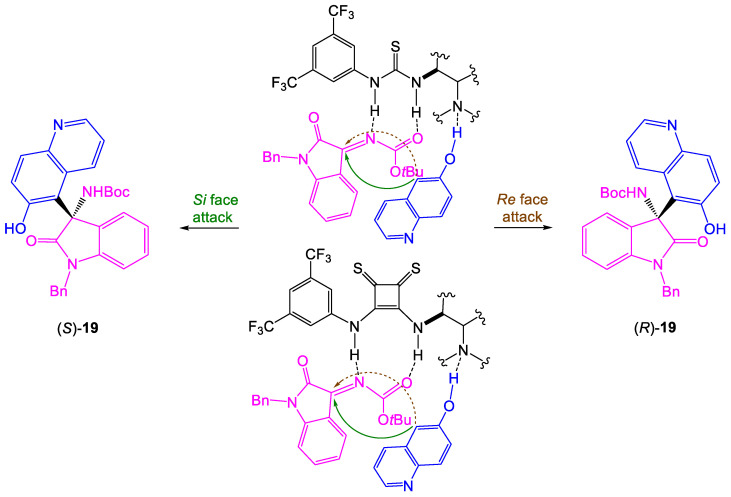
Proposed dual activation for the thiourea and thiosquaramide-catalyzed asymmetric Betti reaction of 6-hydroxyquinoline with ketimine.

Taking into account the obtained results, we have proposed the possible transition state shown in Scheme 5. Bifunctional organocatalysts are responsible for the preorientation and simultaneous activation of electrophile via H-bond donor thiourea/thiosquaramide moiety and the enhancement of the nucleophilicity of 6-hydroxyquinoline by the tertiary amine unit of catalyst [16,17,18]. Generally, nucleophile attacks from C-5 of 6-hydroxyquinoline on the *Si* face of ketimine results in the *S* configuration of product in the case of Takemoto-type organocatalysts **1**–**5** and **9**, while in reactions catalyzed by **6**–**7** and **10**–**11** *Si* face of ketimine becomes less available and the formation of the *R* isomer is favored.

**Scheme 6:**
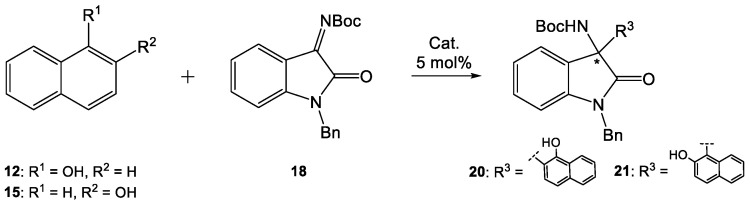
Betti reaction of naphthols **12** and **15** with ketimine **18**.

Finally, the reactions of naphthols **12** and **15** with ketimine **18** were developed (Scheme 6, Table 7). For testing, we chose thiourea **7**, which reacted best with 6-hydroxyquinoline, and its thiosquaramide counterpart **11**. The obtained results, similarly to those obtained for the reaction 6-hydroxyquinoline (**17**) with ketimine **18**, indicated a higher activity of thiourea **7** compared to thiosquaramide **11**. 3-Substituted 3-amino-2-oxindoles were obtained in moderate yield (53%) and enantioselectivity (78% ee) in the presence of **7**, while thiosquaramide **11** led to racemic products. 

## 3. Materials and Methods

Commercially available chemicals used in this work were purchased from Merck (Darmstadt, Germany) and were used as supplied, without additional purification. NMR spectra were recorded in CDCl_3_ on a Bruker Avance III (600 MHz for ^1^H NMR, 150 MHz for ^13^C NMR); coupling constants are reported in hertz (Hz). The chemical shift values were expressed in ppm (part per million) with tetramethylsilane (TMS) as an internal reference. The rotations were measured using an Anton Paar MCP 500 polarimeter. Melting points measured on the DigiMelt apparatus are uncorrected. The milling treatments were carried out in a vibrating Retsch Mixer Mill 400 (vbm). Milling load is defined as the sum of the mass of the reactants per free volume in the jar. All the reactions using a vibratory ball mill were performed at 25 Hz under air with no interruption of the milling. Chromatographic purification of compounds was achieved with 230–400 mesh size silica gel. The progress of reactions was monitored by silica gel thin-layer chromatography plates (Merck TLC Silicagel 60 F254). The enantiomeric excess was determined by HPLC (1260 Infinity, Agilent Technologies, Santa Clara, CA, USA), employing a Chiralpak AD-H or OD-H column (25 cm × 4.6 mm).

Copies of ^1^H and ^13^C NMR spectra of compounds **1**–**3**, **5**–**11**, **14**, **16**, **19**–**21** and selected HPLC chromatograms of Betti bases are included in the Supplementary Materials.

### 3.1. General Procedure for the Synthesis of Thiourea Catalysts

To the solution of corresponding diamine (0.4 mmol) in CH_2_Cl_2_ at 0 °C, isothiocyanate (0.48 mmol) was added dropwisely under argon atmosphere. The resulting mixture was stirred at room temperature for around 30 min. The progress of the reaction was monitored by silica gel thin-layer chromatography plates. After reaction completion, the crude product was concentrated and purified by column chromatography. 

#### 3.1.1. 1-[3,5-Bis(trifluoromethyl)phenyl]-3-[(1*R*,2*R*)-2-(piperidin-1-yl)cyclohexyl]thiourea (**1**)

Yellowish solid, 167 mg, 92% yield; R_f_ = 0.09 (hexane/ethyl acetate, 2:1); [α${]}_{D}^{20}$ = −6.0 (*c* 0.4, CHCl_3_), {Lit. [32]: [α${]}_{D}^{20}$ = 0.60 (*c* 0.5, CHCl_3_)}; m.p. = 53.2–55.5 °C, {Lit. [32]: m.p. = 57–59 °C}; δ_H_ (600 MHz, CDCl_3_): 1.10–1.47 (m, 10H, H_aliph_), 1.75 (d, 1H, *J* = 13.1, H_aliph_), 1.85 (d, 1H, *J* = 11.2, H_aliph_), 1.94 (d, 1H, *J* = 11.5, H_aliph_), 2.33–2.55 (m, 3H, H_aliph_), 2.57–2.82 (m, 3H, H_aliph_), 3.85 (bs, 1H, NH), 7.68 (s, 1H, H_ar_), 7.87 (s, 2H, H_ar_); δ_C_ (150 MHz, CDCl_3_): 23.5, 23.8, 24.4, 25.1, 25.7, 27.2, 32.6 (C_aliph_), 49.7 (CH_2_N), 55.6 (CHNH), 68.6 (CHN), 118.7, 120.3, 122.1, 123.8, 124.1 (q, ^1^*J*_C-F_ = 270.0 Hz), 132.5 (q, ^2^*J*_C-F_ = 34.7 Hz), 140.0 (C_ar_) 180.3 (CS); Elementar analysis: C_20_H_25_F_6_N_3_S (453.49 g/mol) calculated: C% 52.97, H% 5.56, N% 9.27, S% 7.07; found: C% 52.81, H% 5.61, N% 9.47, S% 6.97.

Spectral data matched that reported by Mukherjee [32].

#### 3.1.2. 1-(3,4,5-Trimetoxyphenyl)-3-[(1*R*,2*R*)-2-(piperidin-1-yl)cyclohexyl]thiourea (**2**)

Yellowish solid, 143 mg, 88% yield; R_f_ = 0.12 (hexane/ethyl acetate, 2:1); [α${]}_{D}^{20}$ = −23.80 (*c* 0.5, CHCl_3_); m.p. = 99.3–103.1 °C; δ_H_ (600 MHz, CDCl_3_): 0.80–0.91 (m, 1H, H_aliph_), 1.05–1.14 (m, 1H, H_aliph_), 1.21–1.31 (m, 5H, H_aliph_), 1.32–1.42 (m, 6H, H_aliph_), 1.71 (d, 1H, *J =* 13.8 H_aliph_), 1.83 (d, 1H, *J =* 10.7, H_aliph_), 1.88 (d, 1H, *J =* 11.4, H_aliph_), 2.27 (bs, 2H, CHN), 2.42 (bs, 1H, NH), 2.63 (bs, 2H, CHN), 2.89 (bs, 1H, NH), 3.84 (s, 9H, OCH_3_), 6.57 (s, 2H, H_ar_); δ_C_ (150 MHz, CDCl_3_): 14.2, 23.4, 24.6, 25.6, 29.8, 30.4, 32.7 (C_aliph_), 49.3 (CHN), 55.5 (CHN), 56.5 (2OCH_3_), 61.0 (OCH_3_), 68.3 (CHN), 103.5, 132.7, 137.0, 153.9 (C_ar_), 181.0 (CS); Elementar analysis: C_21_H_33_N_3_O_3_S (407.57 g/mol) calculated: C% 61.89, H% 8.16, N% 10.31, S% 7.87; found: C% 61.79, H% 8.10, N% 10.10, S% 7.75.

#### 3.1.3. 1-[3,5-Bis(trifluoromethyl)phenyl]-3-[(1*R*,2*R*)-2(dimethylamino)cyclohexyl]thiourea (**3**)

Commercially available catalyst (Merck). 

#### 3.1.4. 1-(3,4,5-Trimetoxyphenyl)-3-[(1*R*,2*R*)-2-(dimethylamino)cyclohexyl]thiourea (**4**)

Yellowish solid, 144 mg, 98% yield; R_f_ = 0.24 (hexane/ethyl acetate, 2:1); [α${]}_{D}^{20}$ = −82.17 (*c* 0.5, CHCl_3_); m.p. = 135.5–136.5 °C; δ_H_ (600 MHz, CDCl_3_): 0.95–1.05 (m, 1H, H_aliph_), 1.11–1.27 (m, 3H, H_aliph_), 1.30–1.39 (m, 1H, H_aliph_), 1.68 (d, 1H, *J =* 13.5 Hz, H_aliph_), 1.82 (d, 1H, *J =* 12.8 Hz, H_aliph_), 1.86 (d, 1H, *J =* 12.8 Hz, H_aliph_), 2.23 (s, 6H, 2CH_3_), 2.32–2.41 (ddd, 1H, *J =* 5.5, 5.5, 2.88 Hz, CHN), 2.64–2.74 (m, 1H, CHN), 3.82 (s, 6H, 2OCH_3_), 3.83 (s, 3H, OCH_3_), 3.97 (bs, 1H, NH), 6.50 (s, 2H, H_ar_), 6.91 (bs, 1H, NH); δ_C_ (150 MHz, CDCl_3_): 21.6, 24.6, 25.1, 32.9 (C_aliph_), 40.1 (CH_3_), 56.0 (CHN), 56.3 (2OCH_3_), 61.0 (OCH_3_), 66.8 (CHN), 102.4, 133.2, 136.3, 153.7 (C_ar_), 179.9 (CS); Elementar analysis: C_18_H_29_N_3_O_3_S (367.51 g/mol) calculated: C% 58.83, H% 7.95, N% 11.43, S% 8.72; found: C% 58.64, H% 7.81, N% 11.68, S% 8.56.

Spectral data matched that reported by Steve Tse and Yeung [36].

#### 3.1.5. 1-(Perfluorophenyl)-3-[(1*R*,2*R*)-2-(dimethylamino)cyclohexyl]thiourea (**5**)

Yellowish solid, 144 mg, 78% yield; R_f_ = 0.44 (hexane/ethyl acetate, 2:1); [α${]}_{D}^{20}$ = +101.68 (*c* 0.35, CHCl_3_); m.p. = 117.8–119.5 °C; δ_H_ (600 MHz, CDCl_3_): 0.96–1.30 (m, 5H, H_aliph_), 1.70 (d, 1H, *J =* 9.4 Hz, H_aliph_), 1.77 (m, 1H, *J =* 12.7 Hz, H_aliph_), 1.85 (d, 1H, *J =* 12.7 Hz, H_aliph_), 2.13–2.20 (m, 1H, H_aliph_), 2.28 (s, 6H, 2CH_3_), 2.38–2.47 (m, 1H, H_aliph_), 3.58 (bs, 1H, NH), 6.34 (bs, 1H, NH); δC (150 MHz, CDCl_3_): 20.9, 25.3, 25.8, 33.3 (C_aliph_), 40.3, 40.7 (CH_3_), 57.3, 69.9 (CHN), 137.2, 138.8 (C_ar_), 178.3 (CS); Elementar analysis: C_15_H_18_F_5_N_3_S (367.38 g/mol) calculated: C% 49.04, H% 4.94, N% 11.44, S% 8.73; found: C% 48.89, H% 5.30, N% 10.59, S% 8.76.

#### 3.1.6. 1-[3,5-Bis(trifluoromethyl)phenyl]-3-[(*R*)-4-methyl-1-(piperidin-1-yl)pentan-2-yl]thiourea (**6**)

Colorless solid, 138 mg, 76% yield; R_f_ = 0.21 (hexane/ethyl acetate, 2:1); [α${]}_{D}^{20}$ = −26.20 (*c* 0.4, CHCl_3_), {Lit. [33]: [α${]}_{D}^{20}$ = −30.80 (*c* 1.0, CHCl_3_)}; m.p. = 43.5–45.5 °C; δ_H_ (600 MHz, CDCl_3_): 0.96 (d, 3H, *J =* 5.5 Hz, CH_3_), 0.98 (d, 3H, *J =* 6.7 Hz, CH_3_), 1.29–1.35 (m, 1H, H_aliph_), 1.41–1.67 (m, 7H, H_aliph_), 1.72–1.80 (m, 1H, CH(CH_3_)_2_), 2.30–2.50 (m, 3H, H_aliph_), 2.55–2.85 (m, 3H, H_aliph_), 3.79 (bs, 1H, H_aliph_), 6.23 (s, 1H), 7.64 (s, 1H, H_ar_), 8.05 (s, 2H, H_ar_), 12.77 (bs, 1H, NH); δC (150 MHz, CDCl_3_): 22.2 (C_aliph_), 22.8 (CH_3_), 23.5 (C_aliph_), 25.0 (CH_3_), 25.7 (*C*H(CH_3_)_2_), 31.0, 43.0 (*C*H_2_CH(CH_3_)_2_), 53.0 (CH_2_N), 55.3 (CHN), 67.2 (CH_2_N), 118.3, 122.4, 124.2, 124.8, 126.0 (C_6_H_3_(CF_3_)_2_), 131.8 (q, ^2^*J*_C-F_ = 32.7 Hz), 141.9 (C_ar_), 183.8 (CS); Elementar analysis: C_20_H_27_F_6_N_3_S (455.51 g/mol) calculated: C% 52.74, H% 5.97, N% 9.23, S% 7.04; found: C% 52.66, H% 6.02, N% 9.08, S% 7.02.

Spectral data matched that reported by Qu [33]. 

#### 3.1.7. 1-[3,5-Bis(trifluoromethyl)phenyl]-3-[(*R*)-1-phenyl-3-(piperidin-1-yl)propan-2-yl]thiourea (**7**)

Colorless solid, 161 mg, 82% yield; R_f_ = 0.17 (hexane/ethyl acetate, 2:1); [α${]}_{D}^{20}$ = −30.36 (c 0.4, CHCl_3_); {Lit. [34]: [α${]}_{D}^{20}$ = −32.90 (c 0.65, CH_2_Cl_2_)}; m.p. = 52.5–54.5 °C; δ_H_ (600 MHz, CDCl_3_): 1.34–1.69 (m, 7H, H_aliph_), 2.29 (bs, 2H, H_aliph_), 2.42–2.66 (m, 4H, H_aliph_), 2.72–2.82 (m, 1H, H_aliph_), 2.92–3.04 (m, 1H, H_aliph_), 4.02 (bs, 1H, NH), 6.35 (bs, 1H, NH), 7.22 (d, 2H, *J =* 7.2, H_ar_), 7.27–7.32 (m, 1H, H_ar_), 7.34–7.38 (m, 2H, H_ar_), 7.65 (s, 1H, H_ar_), 8.01 (s, 2H, H_ar_), 12.85 (bs, 1H, NH); δC (150 MHz, CDCl_3_): 23.5, 25.6 (C_aliph_), 40.0 (*C*H_2_C_6_H_5_), 54.8 (2CH_2_N), 55.5 (CHN), 65.1 (CH_2_N), 118.5, 120.6, 122.4, 124.2, 125.0, 126.0, 127.5, 128.9, 129.2, 129.3, 131.0, 131.9 (q, ^2^*J*_C-F_ = 32.7 Hz), 136.2, 141.8 (C_ar_), 183.5 (CS); Elementar analysis: C_23_H_25_F_6_N_3_S (489.52 g/mol) calculated: C% 6.43, H% 5.15, N% 8.58, S% 6.55; found: C% 56.49, H% 5.04, N% 8.64, S% 6.53.

Spectral data matched that reported by Zhu [34].

### 3.2. General Procedure for the Synthesis of Thiosquaramides

Procedure A: The 3-[(3,5-bis(trifluoromethyl)phenyl)amino]-4-(cyclopentyloxy)cyclobut-3-ene-1,2-thione (124.0 mg, 0.3 mmol, 1 eq.) was dissolved under argon atmosphere in dry CH_2_Cl_2_ (1 mL), cooled to 0 °C, then the appropriate amine (0.3 mmol, 1 eq.) was added. The mixture was stirred for about 1 h at 0 °C, while the reaction was monitored by TLC tests. Then, the solvent was evaporated, and the obtained crude product was purified by silica gel column chromatography, using a solvent mixture of ethyl acetate:methanol = 7:1 as the eluent.

Procedure B: The 3-[(3,5-bis(trifluoromethyl)phenyl)amino]-4-(cyclopentyloxy)cyclobut-3-ene-1,2-thione [31] (124.0 mg, 0.3 mmol, 1 eq.) and appropriate amine (0.3 mmol, 1 eq.) were introduced in a 5 mL stainless steel grinding jar with 3 stainless steel balls (5 mm diameter). The jar was closed and subjected to grinding for 35 min in the vibratory ball mill operated at 23 Hz. The progress of the reaction was monitored by silica gel thin-layer chromatography plates and the obtained crude product was purified by silica gel column chromatography, using a solvent mixture of ethyl acetate:methanol = 7:1 as the eluent.

A detailed analysis of the resulting structure was performed for the hydrochlorides.

#### 3.2.1. 3-[(3,5-Bis(trifluoromethyl)phenyl)amino]-4-[((1*R*,2*R*)-2-(piperidin-1-yl)cyclohexyl)amino]cyclobut-3-ene-1,2-dithione (**8**)

Yellow–orange solid, 112 mg, 69% yield (procedure A), 138 mg, 85% yield (procedure B); R_f_ = 0.80 (ethyl acetate/methanol, 7:1); [α${]}_{D}^{20}$ = −34.14 (*c* 0.1, CHCl_3_); m.p. = 137.5–139.8 °C {Lit. [31]: m.p. = 138–144 °C}; NMR characterization for 8·HCl: δ_H_ (600 MHz, CDCl_3_): 1.35–1.63 (m, 4H, H_aliph_), 1.84–2.11 (m, 7H, H_aliph_), 2.15–2.25 (m, 1H, H_aliph_), 2.48–2.59 (m, 2H, H_aliph_), 2.76–2.87 (m, 1H, H_aliph_), 3.06–3.16 (m, 1H, H_aliph_), 3.34–3.45 (m, 2H, CHN), 3.46–3.54 (m, 1H, CHN), 5.09–5.19 (m, 1H, CHN), 7.63 (s, 1H, H_ar_), 8.23 (s, 2H, H_ar_), 9.26 (bs, 1H, NH), 9.46 (bs, 1H, NH), 10.51 (bs, 1H, NH); δ_C_ (150 MHz, CDCl_3_): 22.3, 23.2, 23.6, 24.0, 24.5, 29.8, 35.7 (C_aliph_), 47.4 (2CH_3_), 53.2, 53.7 (CHN), 68.2, 118.4, 121.9, 122.2, 124.0, 125.8 (C_6_H_3_(CF_3_)_2_), 132.3 (q, ^2^*J*_C-F_ = 33.6 Hz), 137.8 (C_ar_), 169.4, 170.4 (C=C), 206.3, 209.4 (CS); TOF MS ES^+^ calculated for C_23_H_25_N_3_F_6_S_2_ [M]^+^ 522.1472; found 522.1475.

Spectral data matched that reported by Rawal [31].

#### 3.2.2. 3-[(3,5)-Bis(trifluoromethyl)phenyl)amino]-4-[((1*R*,2*R*)-2-(dimethylamino)cyclohexyl)amino]cyclobut-3-ene-1,2-dithione (**9**)

Yellow–orange solid, 112 mg, 74% yield (procedure A), 134 mg, 89% yield (procedure B); R_f_ = 0.82 (ethyl acetate/methanol, 7:1); [α${]}_{D}^{20}$ = −28.82 (*c* 0.3, CHCl_3_); m.p. = 153.2–156.0 °C {Lit. [31]: m.p. = 156–160 °C}; NMR characterization for 9·HCl: δ_H_ (600 MHz, CDCl_3_): 0.85–0.91 (m, 1H, H_aliph_), 1.83–1.91 (m, 2H, H_aliph_), 2.00–2.04 (m, 1H, H_aliph_), 2.09–2.14 (m, 1H, H_aliph_), 2.40–2.47 (m, 1H, H_aliph_), 2.74–2.82 (m, 2H, H_aliph_), 2.85 (s, 3H, CH_3_), 2.93 (m, 3H, CH_3_), 3.28–3.36 (m, 1H, H_aliph_), 5.23–5.29 (m, 1H, CHN), 7.63 (s, 1H, H_ar_), 8.26 (s, 2H, H_ar_), 9.03 (bs, 1H, NH), 10.30 (bs, 1H, NH), 10.79 (bs, 1H, NH); δ_C_ (150 MHz, CDCl_3_): 23.0, 23.6, 23.7, 34.5, 37.5 (C_aliph_), 43.4 (2CH_3_), 52.9 (CHN), 68.1, 114.3, 118.1, 121.8, 122.0, 123.9, 125.7 (C_6_H_3_(CF_3_)_2_), 132.2 (q, ^2^*J*_C-F_ = 33.5 Hz), 138.0 (C_ar_), 169.6, 171.6 (C=C), 205.2, 209.1 (CS); TOF MS ES^+^ calculated for C_26_H_26_N_3_F_6_S_2_ [M]^+^ 558.1472; found 558.1468. TOF MS ES^+^ calculated for C_20_H_21_N_3_F_6_S_2_ [M]^+^ 482.1159; found 482.1160.

Spectral data matched that reported by Rawal [31].

#### 3.2.3. 3-[(3,5)-Bis(trifluoromethyl)phenyl)amino]-4-[((*S*)-4-methyl-1-(piperidin-1-yl)pentan-2-yl)amino]cyclobut-3-ene-1,2-dithione (**10**)

Yellow–orange solid, 114 mg, 70% yield (procedure A), 140 mg, 86% yield (procedure B); R_f_ = 0.75 (ethyl acetate/methanol, 7:1); [α${]}_{D}^{20}$ = −81.0 (*c* 0.2, CHCl_3_); m.p. = 153.9–156.5 °C; NMR characterization for 10·HCl: δ_H_ (600 MHz, CDCl_3_): 0.97 (d, 3H, *J =* 7.0, CH_3_), 0.98 (d, 3H, *J =* 7.0, CH_3_), 1.37–1.49 (m, 2H, H_aliph_), 1.68–1.77 (m, 2H, H_aliph_), 1.83–1.93 (m, 4H, H_aliph_), 2.10–2.20 (m, 1H, H_aliph_), 2.78–2.91 (m, 2H, H_aliph_), 2.95–3.06 (m, 1H, H_aliph_), 3.15–3.32 (m, 1H, H_aliph_), 3.45–3.58 (m, 1H, H_aliph_), 4.00–4.12 (m, 1H, H_aliph_), 5.63 (bs, 1H, CHN), 7.62 (s, 1H, H_ar_), 8.33 (s, 2H, H_ar_), 9.15 (bs, 1H, NH), 9.84 (bs, 1H, NH), 11.17 (bs, 1H, NH); δ_C_ (150 MHz, CDCl_3_): 21.7, 22.3, 22.5 (C_aliph_), 22.7 (CH_3_), 22.9 (C_aliph_), 24.8 (CH_3_), 43.7 (CH_2_), 48.0 (CHN), 53.4, 56.1, 60.8 (CH_2_), 114.2, 118.2, 122.2, 123.9, 125.7, 129.0 (C_6_H_3_(CF_3_)_2_), 132.1 (q, ^2^*J*_C-F_ = 34.0 Hz), 137.9 (C_ar_), 169.1, 171.3, (C=C), 204.8, 209.0 (CS); TOF MS ES^+^ calculated for C_23_H_27_N_3_F_6_S_2_ [M]^+^ 524.1629; found 524.1631.

#### 3.2.4. 3-[(3,5)-Bis(trifluoromethyl)phenyl)amino]-4-[((*S*)-1-phenyl-3-(piperidin-1-yl)propan-2-yl)amino]cyclobut-3-ene-1,2-dithione (**11**)

Yellow–orange solid, 132 mg, 76% yield (procedure A), 157 mg, 91% yield (procedure B); R_f_ = 0.70 (ethyl acetate/methanol, 7:1); [α${]}_{D}^{20}$ = −35.9 (*c* 0.2, CHCl_3_); m.p. = 159.3–163.0 °C; NMR characterization for 11·HCl: δ_H_ (600 MHz, CDCl_3_): 1.38–1.44 (m, 1H, H_aliph_), 1.69–1.90 (m, 3H, H_aliph_), 1.94–2.02 (m, 1H, H_aliph_), 2.11–2.18 (m, 1H, H_aliph_), 2.63–2.70 (m, 1H, H_aliph_), 2.83–2.91 (m, 2H, H_aliph_), 2.96–3.04 (m, 1H, H_aliph_), 3.43–3.49 (m, 1H, H_aliph_), 3.50–3.57 (m, 1H, H_aliph_), 3.63–3.70 (m, 1H, H_alph_), 3.76–3.84 (m, 1H, H_aliph_), 5.77 (bs, 1H, CHN), 7.30–7.35 (m, 5H, H_ar_), 7.58 (s, 1H, H_ar_), 8.27 (s, 2H, H_ar_), 9.65 (bs, 1H, NH), 9.81 (bs, 1H, NH), 11.04 (bs, 1H, NH); δ_C_ (150 MHz, CDCl_3_): 21.8, 22.9 (C_aliph_), 42.2 (CH_2_N), 50.8 (CHN), 53.2 (CH_2_N), 56.6 (CH_2_N), 59.4 (CH_2_N), 118.5, 121.9, 122.2, 124.0, 128.0, 129.3, 129.4 (C_ar_), 132.2 (q, ^2^*J*_C-F_ = 34.0 Hz), 135.0 (C_6_H_3_(CF_3_)_2_), 137.9, 169.0 (C=C), 171.2, 209.0, 209.8 (CS); TOF MS ES^+^ calculated for C_26_H_26_N_3_F_6_S_2_ [M]^+^ 558.1472; found 558.1468.

### 3.3. General Procedure for the Reaction of N-tosylimine (**13**) with 1- and *2-naphthol (**12** or **15**) in the Presence of Catalyst **1**–**11***

22.0 mg of 1- or 2-naphthol (0.15 mmol, 3 eq.), 13.0 mg of *N*-benzylidene-*para*-toluenesulfonamide (13) (0.05 mmol, 1 eq.), 0.005 mmol (10 mol%) of a corresponding organocatalyst, 4 Å molecular sieves, and 1 mL of toluene were placed in the Schlenk flask. The reaction was carried out under an argon atmosphere at 0 °C and the progress of the reaction was controlled by TLC tests. After completion of the reaction, the solvent was evaporated, and the residue was purified by silica gel column chromatography using a solvent mixture of hexane:ethyl acetate = 4:1 as eluent. 

#### 3.3.1. *N*-[(1-hydroxynaphtalen-2-yl)(phenyl)methyl]-4-methylbenzene sulfonamide (**14**)

Colorless oil, 20 mg, 98% yield; R_f_ = 0.29 (hexane/ethyl acetate, 4:1); [α${]}_{D}^{20}$ = −30.2 (*c* 0.9; CH_2_Cl_2_) for ee = 80% (Table 1, entry 6) {Lit. [14]: [α${]}_{D}^{22}$ = −38.8 (c 1.0; CH_2_Cl_2_ for ee = 94%)}; δ_H_ (600 MHz, CDCl_3_): 2.18 (s, 3H, CH_3_), 5.91 (s, 1H, CH), 6.89 (d, 2H, *J =* 8.5), 6.97 (d, 2H, *J =* 8.0), 7.18–7.21 (m, 2H), 7.22–7.28 (m, 3H), 7.45–7.48 (m, 2H), 7.53–7.56 (m, 2H), 7.71–7.74 (m, 1H), 8.03–8.07 (m, 1H); δ_C_ (150 MHz, CDCl_3_): 21.3, 58.7, 119.5, 120.6, 121.3, 125.3, 125.6, 126.2, 126.6, 127.1, 127.2, 127.6, 127.8, 128.7, 129.2, 134.2, 136.1, 139.0, 143.7, 149.5.

Spectral data matched that reported by Wang [14].

HPLC: Kromasil AD-H (250 × 4.6 mm), hexane/*i*PrOH = 90/10, flow = 1 mL/min, *λ* = 235 nm, *t*_S_ = 26.2 min and *t*_R_ = 31.7 min.

#### 3.3.2. *N*-[(2-hydroxynaphtalen-1-yl)(phenyl)methyl]-4-methylobenzene sulfonamide (**16**)

Colorless solid, 20 mg, 98% yield; R_f_ = 0.32 (hexane/ethyl acetate, 4:1); m.p. = 139.0–140.9 °C {Lit. [13]: m.p. = 140–142 °C}; [α${]}_{D}^{20}$ = +33.4 (*c* 0.5, CHCl_3_) for ee = 54% (Table 3, entry 2) {Lit. [13]: [α${]}_{D}^{25}$ = +50.0 (*c* 0.26; CHCl_3_ for ee = 84%)}; δ_H_ (600 MHz, CDCl_3_): 2.07 (s. 3H, CH_3_), 5.68 (s, 1H, CH), 6.23 (d, 1H, *J =* 9.2), 6.38 (d, 1H, *J =* 9.2), 6.63 (d, 2H, *J =* 8.0), 6.73 (d, 1H, *J =* 8.7), 7.12–7.18 (m, 3H), 7.22–7.29 (m, 5H), 7.32–7.37 (m, 1H), 7.49 (d, 1H, *J =* 8.8), 7.60 (d, *J =* 8.0 Hz), 7.68 (d, 1H, *J =* 8.5); δ_C_ (150 MHz, CDCl_3_): 21.2. 54.3, 117.7, 118.1, 122.0, 123.4, 126.7, 126.8, 127.2, 127.4, 128.4, 128.7, 129.0, 129.7, 132.4, 136.3, 140.0, 142.8, 151.1.

Spectral data matched that reported by Hui [11].

HPLC: Chiralpak OD-H (250 × 4.6 mm), hexane/*i*PrOH = 94/6, flow = 1 mL/min, *λ* = 235 nm, *t*_S_ = 17.3 min, and *t*_R_ = 22.9 min.

### 3.4. General Procedure for the Reaction of Ketimine (**18**) with 6-hydroxyquinoline (**17**) and Ketimine (**18**) with 1- and *2-naphthol (**12** or **15**)* in the Presence of Catalyst **1**–**11**

14.5 mg (0.1 mmol, 1 eq.) of 6-hydroxyquinoline or 14.4 mg (0.1 mmol, 1 eq.) of corresponding naphthol, 33.6 mg (0.1 mmol, 1 eq.) of ketimine, 0.005 mmol (5 mol%) of the appropriate organocatalyst and 4 Å molecular sieves were placed in the Schlenk flask and diluted with 1 mL of toluene. The reaction was carried out under an argon atmosphere at room temperature (or 0 °C) and the progress of the reaction was controlled by TLC tests. After completion of the reaction, the solvent was evaporated, and the residue was purified by silica gel column chromatography using a solvent mixture of hexane:ethyl acetate = 1:5 as eluent. 

#### 3.4.1. [1-Benzyl-3-(6-hydroxyquinolin-5-yl)-2-oxoindolin-3-yl]carbamate tert-butyl (**19**)

Yellowish solid, 37.5 mg, 78% yield; R_f_ = 0.26 (hexane/ethyl acetate, 1:5); m.p. = 180.5–183.0 °C {Lit. [17]: m.p. = 183–186 °C}; [α${]}_{D}^{20}$ = −366.3 (*c* 0.3, MeOH) for ee = 98% (Table 6, entry 9) {Lit. [17]: [α${]}_{D}^{20}$ = −368.7 (*c* 0.31; MeOH for ee = 98%)}; δ_H_ (600 MHz, CDCl_3_): 1.32 (s, 9H, C(CH_3_)_3_), 4.86 (d, 1H, *J =* 16.1, CH_2_Ph), 5.21 (d, 1H, *J =* 16.1, CH_2_Ph), 5.82 (s, 1H), 6.71–6.80 (m, 2H), 6.85 (d, 1H, *J =* 7.9), 7.08–7.12 (m, 1H), 7.19–7.25 (m, 4H), 7.27–7.32 (m, 2H), 7.34–7.38 (m, 1H), 7.48 (d, 1H, *J =* 9.1), 8.00 (d, 1H, *J =* 9.1), 8.57 (dd, 1H, *J =* 4.0, 1.3), 8.77 (dd, 1H), 10.45 (s, 1H, OH); δ_C_ (150 MHz, CDCl_3_): 28.2 (CH_3_), 44.7 (CH_2_), 65.1, 80.9, 110.1, 114.4, 119.9, 123.4, 124.8, 125.8, 127.2, 127.5, 127.7, 129.7, 130.1, 132.2, 132.7, 135.2, 143.2, 144.4, 146.4, 153.9, 155.8, 178.8.

Spectral data matched that reported by Pedro [17].

HPLC: Kromasil AD-H (250 × 4,6 mm), eluent hexane/*i*PrOH = 80/20, flow = 1 mL/min, *λ* = 235 nm, *t*_S_ = 12.9 min and *t*_R_ = 31.7 min.

#### 3.4.2. [1-Benzyl-3-(1-hydroxynaphthalen-2-yl)-2-oxoindolin-3-yl)carbamate tert-butyl (**20**)

Colorless solid, 25.4 mg, 53% yield; R_f_ = 0.55 (hexane/ethyl acetate, 2:1); m.p. = 102.4–103.9 °C {Lit. [15]: m.p. = 104–105 °C}; [α${]}_{D}^{20}$ = +284.7 (*c* 0.4, CHCl_3_) for ee = 78% (Table 7, entry 1) {Lit. [15]: [α${]}_{D}^{20}$ = +359.6 (*c* 0.44, CHCl_3_ for ee = 99%)}; δ_H_ (600 MHz, CDCl_3_): 1.32 (s, 9H, C(CH_3_)_3_), 4.84 (s, 1H, CH_2_Ph), 5.06 (d, 1H, *J =* 12.0, CH_2_Ph), 5.79 (s, 1H), 6.77 (d, 1H, *J* = 7.8), 6.81 (d, 1H, *J =* 8.7), 7.16–7.27 (m, 7H), 7.28–7.34 (m, 1H), 7.41 (d, 1H, *J =* 7.3), 7.48–7.55 (m, 2H), 7.68–7.75 (m, 1H), 8.44–8.50 (m, 1H), 10.79 (s, 1H, OH); δ_C_ (150 MHz, CDCl_3_): 28.3 (CH_3_), 44.6 (CH_2_), 65.3, 80.9, 108.7, 110.5, 114.7, 119.8, 123.4, 123.6, 125.2, 125.5, 125.8, 127.2, 127.3, 127.6, 127.8, 128.9, 129.6, 135.0, 135.1, 142.8, 154.1, 155.4, 180.0.

Spectral data matched that reported by Pedro [15].

HPLC: Kromasil AD-H (250 × 4.6 mm), eluent hexane/*i*PrOH = 80/20, flow = 1.5 mL/min, *λ* = 254 nm, *t*_S_ = 10.5 min and *t*_R_ = 49.8 min.

#### 3.4.3. [1-Benzyl-3-(2-hydroxynaphthalen-1-yl)-2-oxoindolin-3-yl)carbamate tert-butyl (**21**)

Colorless solid, 23.5 mg, 49% yield; R_f_ = 0.42 (hexane/ethyl acetate, 2:1); m.p. = 140.2–141.7 °C {Lit. [20]: m.p. = 140.5–142.0 °C}; [α${]}_{D}^{20}$ = −7.6 (*c* 0.4, CHCl_3_) for ee = 46% (Table 7, entry 3) {Lit. [20]: [α${]}_{D}^{25}$ = −15.6 (*c* 0.50, CHCl_3_ for ee = 93%)}; δ_H_ (600 MHz, CDCl_3_): 1.31 (s, 9H, C(CH_3_)_3_), 4.87 (d, *J =* 16.0 Hz, 1H, CH_2_Ph), 5.11 (d, *J =* 16.0 Hz, 1H, CH_2_Ph), 5.90 (s, 1H), 6.76 (d, 1H, *J* = 7.8), 7.00 (d, 1H, *J =* 7.7 Hz), 7.05–7.12 (m, 2H), 7.18–7.27 (m, 6H), 7.31–7.37 (m, 3H), 7.53 (d, 1H, *J* = 8.7), 7.66 (d, 1H, *J* = 7.5), 9.76 (s, 1H, OH); δ_C_ (150 MHz, CDCl_3_): 28.3 (CH_3_), 44.8 (CH_2_), 65.5, 80.8, 110.1, 114.8, 121.2, 123.0, 123.5, 124.6, 125.4, 125.8, 127.7, 128.9, 130.4, 130.7, 131.6, 132.0, 135.3, 143.2, 154.0, 155.3, 179.8.

Spectral data matched that reported by Jin [20].

HPLC: Kromasil AD-H (250 × 4.6 mm), eluent hexane/*i*PrOH = 80/20, flow = 1.5 mL/min, *λ* = 254 nm, *t*_S_ = 10.6 min, and *t*_R_ = 21.7 min.

## 4. Conclusions

In conclusion, we have developed an enantioselective Betti reaction of 1- and 2-naphthols and 6-hydroxyquinoline with tosylimine and ketimine compounds using thioureas and, for the first time, thiosquaramide bifunctional organocatalysts. 

We obtained synthetically and medicinally useful chiral aminoarylnaphthols, in excellent yield (up to 98%) with high enantioselectivity (up to 80% ee) and enantioenriched 3-amino-2-oxindoles (up to 78% yield, up to 98% ee). Moreover, we have shown that Takemoto-type thioureas most efficiently catalyze the reaction of 1-naphthol with *N*-tosyl imine (80% ee), while in the reaction of 2-naphthol with *N*-tosyl imine, the bifunctional thiosquaramides organocatalysts demonstrated higher catalytic activity (up to 71% for thiosquaramide **8** vs. 61% ee for thiourea **6**). The reaction of 6-hydroxyquinoline with isatin-derived *N*-Boc ketimine led regioselectively to the alkylated at C-5 chiral product with moderate yield and enantioselectivity, and thiourea **7**, which lacked the cyclohexylamine moiety and performed best under these conditions (up to 98% ee) while its thiosquaramide counterpart generated up to 10% ee. Similar observations were made for the reactions of 1- and 2-naphthols with 6-hydroxyquinoline. 

## Figures and Tables

**Table 1:** Screening of reaction conditions of 1-naphthol (**12**) with *N*-tosylimine **13** catalyzed by thiourea **1**.

Entry	1-Naphthol (eq.)	*N*-tosylimine (eq.)	mol% Cat.	Solvent	Temp. [°C]	Yield [%] ^1^	ee [%] ^2^ (*Conf*.) ^3^
1	5	1	10	toluene	20	87	68 (*S*)
2	3	1	10	toluene	20	98	75 (*S*)
3	1.5	1	10	toluene	20	85	66 (*S*)
4	1	1.5	10	toluene	20	80	66 (*S*)
5	1	3	10	toluene	20	92	66 (*S*)
6	3	1	10	toluene	0	98	80 (*S*)
7	3	1	5	toluene	20	85	68 (*S*)
8	3	1	30	toluene	20	80	60 (*S*)
9	3	1	10	THF	20	20	46 (*S*)
10	3	1	10	ACN	20	35	44 (*S*)
11	3	1	10	*o*-xylene	20	94	68 (*S*)
12	3	1	10	DCM	20	70	64 (*S*)

Reagents and conditions: 1-naphthol (0.15 mmol, 3 eq.), *N*-tosylimine (0.05 mmol, 1 eq.), solvent (1 mL), molecular sieves 4 Å, 20 h. ^1^ Isolated product. ^2^ Determined by HPLC analysis, Chiralpak AD-H, hexane:*i*PrOH 90:10, 1 mL/min, 235 nm. ^3^ The configuration was established by comparison with the optical rotation from the literature [14,35].

**Table 2:** Asymmetric Betti reaction of 1-naphthol (**12**) with *N*-tosylimine **13** catalyzed by thioureas **1**–**7** or thiosquaramides **8**–**11**.

Entry	Catalyst	Yield [%] ^1^	ee [%] ^2^ (*Conf.*) ^3^
1	**1**	98	80 (*S*)
2	**2**	86	50 (*S*)
3	**3**	98	68 (*S*)
4	**4**	93	62 (*S*)
5	**5**	95	64 (*S*)
6	**6**	80	58 (*R*)
7	**7**	98	60 (*R*)
8	**8**	75	56 (*S*)
9	**8**·HCl	53	40 (*S*)
10	**9**	80	42 (*S*)
11	**9**·HCl	30	30 (*S*)
12	**10**	70	20 (*R*)
13	**10**·HCl	45	16 (*R*)
14	**11**	72	36 (*R*)
15	**11**·HCl	50	28 (*R*)

Reagents and conditions: 1-naphthol (0.015 mmol, 3 eq.), *N*-tosylimine (0.05 mmol, 1 eq.), 10 mol% cat., toluene (1 mL), molecular sieves 4 Å, 0 °C, 20 h. ^1^ Isolated product. ^2^ Determined by HPLC analysis, Chiralpak AD-H, hexane:*i*PrOH 90:10, 1 mL/min, 235 nm. ^3^ The configuration was established by comparison with the optical rotation from the literature [14,35].

**Table 3:** Optimization of the reaction conditions of 2-naphtol (**15**) with *N*-tosylimine **13** catalyzed by thiourea **1**.

Entry	2-Naphthol (eq.)	*N*-tosylimine (eq.)	mol% Cat.	Temp. [°C]	Yield [%] ^1^	ee [%] ^2^ (*Conf.*) ^3^
1	5	1	10	0	90	34 (*S*)
2	3	1	10	0	98	54 (*S*)
3	3	1	10	20	95	24 (*S*)
4	1.5	1	10	0	85	24 (*S*)
5	3	1	5	0	90	52 (*S*)
6	3	1	30	0	87	42 (*S*)

Reagents and conditions: 2-naphthol (0.15 mmol, 3 eq.), *N*-tosylimine (0.05 mmol, 1 eq.), toluene (1 mL), molecular sieves 4 Å, 20 h. ^1^ Isolated product. ^2^ Determined by HPLC analysis, Chiralpak OD-H, hexane:*i*PrOH 94:6, 1 mL/min, 235 nm. ^3^ The configuration was established by comparison with the optical rotation from the literature [11,13].

**Table 4:** Asymmetric Betti reaction of 2-naphthol (**15**) with *N*-tosylimine **13** catalyzed by thioureas **1**–**7** or thiosquaramides **8**–**11**.

Entry	Catalyst	Yield [%] ^1^	ee [%] ^2^ (*Conf.*) ^3^
1	**1**	98	54 (*S*)
2	**2**	65	34 (*S*)
3	**3**	78	26 (*R*)
4	**4**	95	6 (*S*)
5	**5**	65	6 (*S*)
6	**6**	70	61 (*R*)
7	**7**	70	24 *(R)*
8	**8**	65	71 *(S)*
9	**8**·HCl	40	64 *(S)*
10	**9**	50	52 *(S)*
11	**9**·HCl	60	56 *(S)*
12	**10**	40	61 *(R)*
13	**10**·HCl	25	46 *(R)*
14	**11**	30	48 *(R)*
15	**11**·HCl	30	46 *(R)*

Reagents and conditions: 2-naphthol (0.15 mmol, 3 eq.), *N*-tosylimine (0.05 mmol, 1 eq.), 10 mol% cat., toluene (1 mL), molecular sieves 4 Å, 0 °C, 20 h. ^1^ Isolated product. ^2^ Determined by HPLC analysis, Chiralpak OD-H, hexane:*i*PrOH 94:6, 1 mL/min, 235 nm. ^3^ The configuration was established by comparison with the optical rotation from the literature [11,13].

**Table 5:** Optimization of the reaction conditions of 6-hydroxyquinoline (**17**) with ketimine **18** catalyzed by thiourea **1**.

Entry	mol% Cat.	Solvent	Temp. [°C]	Yield [%] ^1^	ee [%] ^2^ (*Conf.*) ^3^
1	5	toluene	20	68	76 (*S*)
2	5	toluene	0	60	87 (*S*)
3	5	DCM	20	45	16 (*S*)
4	10	toluene	20	65	50 (*S*)
5	30	toluene	20	60	71 (*S*)

Reagents and conditions: 6-hydroxyquinoline (0.1 mmol, 1 eq.), ketimine (0.1 mmol, 1 eq.), solvent (1 mL), molecular sieves 4 Å, 48 h. ^1^ Isolated product. ^2^ Determined by HPLC analysis, Chiralpak AD-H, hexane:*i*PrOH 80:20, 1 mL/min, 254 nm. ^3^ The configuration was established by comparison with the optical rotation from the literature [17].

**Table 6:** Asymmetric Betti reaction of 6-hydroxyquinoline (**17**) with ketimine **18** catalyzed by thioureas **1**–**7** or thiosquaramides **8**–**11**.

Entry	Catalyst	Temp. [°C]	Yield [%] ^1^	ee [%] ^2^ (*Conf.*) ^3^
1	**1**	20	68	76 (*S*)
2	**1**	0	60	87 (*S*)
3	**2**	20	55	66 (*S*)
4	**3**	20	65	92 (*S*) ^4^
5	**4**	20	55	74 (*S*)
6	**5**	20	68	66 (*S*)
7	**6**	20	68	76 (*R*)
8	**7**	20	78	90 (*R*)
9	**7**	0	75	98 (*R*)
10	**8**	20	50	8 (*R*)
11	**8**·HCl	20	35	12 (*R*)
12	**9**	20	50	12 (*S*)
13	**9**·HCl	20	40	14 (*S*)
14	**10**	20	65	2 (*R*)
15	**10**·HCl	20	45	6 (*R*)
16	**11**	20	65	10 (*R*)
17	**11**·HCl	20	40	2 (*R*)

Reagents and conditions: 6-hydroxyquinoline (0.1 mmol, 1 eq.), ketimine (0.1 mmol, 1 eq.), 5 mol% cat., toluene (1 mL), molecular sieves 4 Å, 48 h. ^1^ Isolated product. ^2^ Determined by HPLC analysis, Chiralpak AD-H, hexane:*i*PrOH 80:20, 1 mL/min, 235 nm. ^3^ The configuration was established by comparison with the optical rotation from the literature [17]. ^4^ Lit. [17] ee = 93%.

**Table 7:** Asymmetric Betti reaction of 1-naphthol (**12**) and ***2***-naphthol (**15**) with ketimine **18** catalyzed by thiourea **7** or thiosquaramide **11**.

Entry	Naphthol	Catalyst	Yield [%] ^1^	ee [%] ^2^ (*Conf.*) ^3^
1	**12**	**7**	53	78 (*R*)
2	**12**	**11**	30	2 (*S*)
3	**15**	**7**	49	46 (*R*)
4	**15**	**11**	33	4 (*S*)

Reagents and conditions: naphthol (0.1 mmol, 1 eq.), ketimine (0.1 mmol, 1 eq.), 5% mol cat., toluene (1 mL), molecular sieves 4 Å, 20 °C, 48 h. ^1^ Isolated product. ^2^ Determined by HPLC analysis, Chiralpak AD-H, hexane:*i*PrOH 80:20, 1.5 mL/min, 254 nm. ^3^ The configuration was established by comparison with the optical rotation from the literature [15,18,19,20].

## Data Availability

The data presented in this study are available in the main text of this article/Supplementary Materials of this article or on request from the corresponding author.

## Supplementary Materials

The following supporting information can be downloaded at: https://www.mdpi.com/article/10.3390/molecules28237835/s1: Figure S1. ^1^H NMR (600 MHz, CDCl_3_) spectrum of **1**; Figure S2. ^13^C NMR (150 MHz, CDCl_3_) spectrum of **1**; Figure S3. ^1^H-^1^H COSY spectrum of **2**; Figure S4. ^1^H-^13^C HMQC spectrum of **2**; Figure S5. ^1^H NMR (600 MHz, CDCl_3_) spectrum of **4**; Figure S6. ^13^C NMR (150 MHz, CDCl_3_) spectrum of **4**; Figure S7. ^1^H NMR (600 MHz, CDCl_3_) spectrum of **5**; Figure S8. ^13^C NMR (150 MHz, CDCl_3_) spectrum of **5**; Figure S9. ^1^H NMR (600 MHz, CDCl_3_) spectrum of **6**; Figure S10. ^13^C NMR (150 MHz, CDCl_3_) spectrum of **6**; Figure S11. ^1^H NMR (600 MHz, CDCl_3_) spectrum of **7**; Figure S12. ^13^C NMR (150 MHz, CDCl_3_) spectrum of **7**; Figure S13. ^1^H NMR (600 MHz, CDCl_3_) spectrum of **8**; Figure S14. ^13^C NMR (150 MHz, CDCl_3_) spectrum of **8**; Figure S15. ^1^H NMR (600 MHz, CDCl_3_) spectrum of **9**; Figure S16. ^13^C NMR (150 MHz, CDCl_3_) spectrum of **9**; Figure S17. ^1^H NMR (600 MHz, CDCl_3_) spectrum of **10**; Figure S18. ^13^C NMR (150 MHz, CDCl_3_) spectrum of **10**; Figure S19. ^1^H NMR (600 MHz, CDCl_3_) spectrum of **11**; Figure S20. ^13^C NMR (150 MHz, CDCl_3_) spectrum of **11**; Figure S21. ^1^H NMR (600 MHz, CDCl_3_) spectrum of **14**; Figure S22. ^13^C NMR (150 MHz, CDCl_3_) spectrum of **14**; Figure S23. ^1^H NMR (600 MHz, CDCl_3_) spectrum of **16**; Figure S24. ^13^C NMR (150 MHz, CDCl_3_) spectrum of **16**; Figure S25. ^1^H NMR (600 MHz, CDCl_3_) spectrum of **19**; Figure S26. ^13^C NMR (150 MHz, CDCl_3_) spectrum of **19**; Figure S27. ^1^H NMR (600 MHz, CDCl_3_) spectrum of **20**; Figure S28. ^13^C NMR (150 MHz, CDCl_3_) spectrum of **20**; Figure S29. ^1^H NMR (600 MHz, CDCl_3_) spectrum of **21**; Figure S30. ^13^C NMR (150 MHz, CDCl_3_) spectrum of **21**. Copies of selected HPLC chromatograms of Betti bases.
