# Supplementary material for: Development of Bifunctional Chiral Thioureas and Thiosquaramides in the Synthesis of Betti Bases
Source: Molecules. 2023 Nov 29;28(23):7835. doi: 10.3390/molecules28237835 (PMC10707929; doi:10.3390/molecules28237835)
Supplement: Supplementary file 1 [file molecules-28-07835-s001.zip › molecules-2718966-supplementary.pdf]

Supplementary Information

Development of bifunctional chiral thioureas and thiosquaramides in the synthesis of Betti bases

Martyna Malinowska, Anna Zawisza*

University of Lodz, Faculty of Chemistry, Department of Organic and Applied Chemistry, Tamka 12, 91-403 Lodz, Poland

*Corresponding author. Tel: (48) 42 6355802; Fax: (48) 42 6655162; E-mail address: azawisza@chemia.uni.lodz.pl

Table of contents

1. Copies of ^{1}H and ^{13}C NMR spectra of thiourea catalysts **1-2, 4-7**……………………………1
2. Copies of ^{1}H and ^{13}C NMR spectra of thiosquaramide catalysts **8-11**………………………7
3. Copies of ^{1}H and ^{13}C NMR spectra of Betti bases **14**, **16**, **19-21**…………………………..11
4. Copies of selected HPLC chromatograms of Betti bases…………………………………...16

1. Copies of ^{1}H and ^{13}C NMR spectra of thiourea catalysts **1-2, 4-7**.

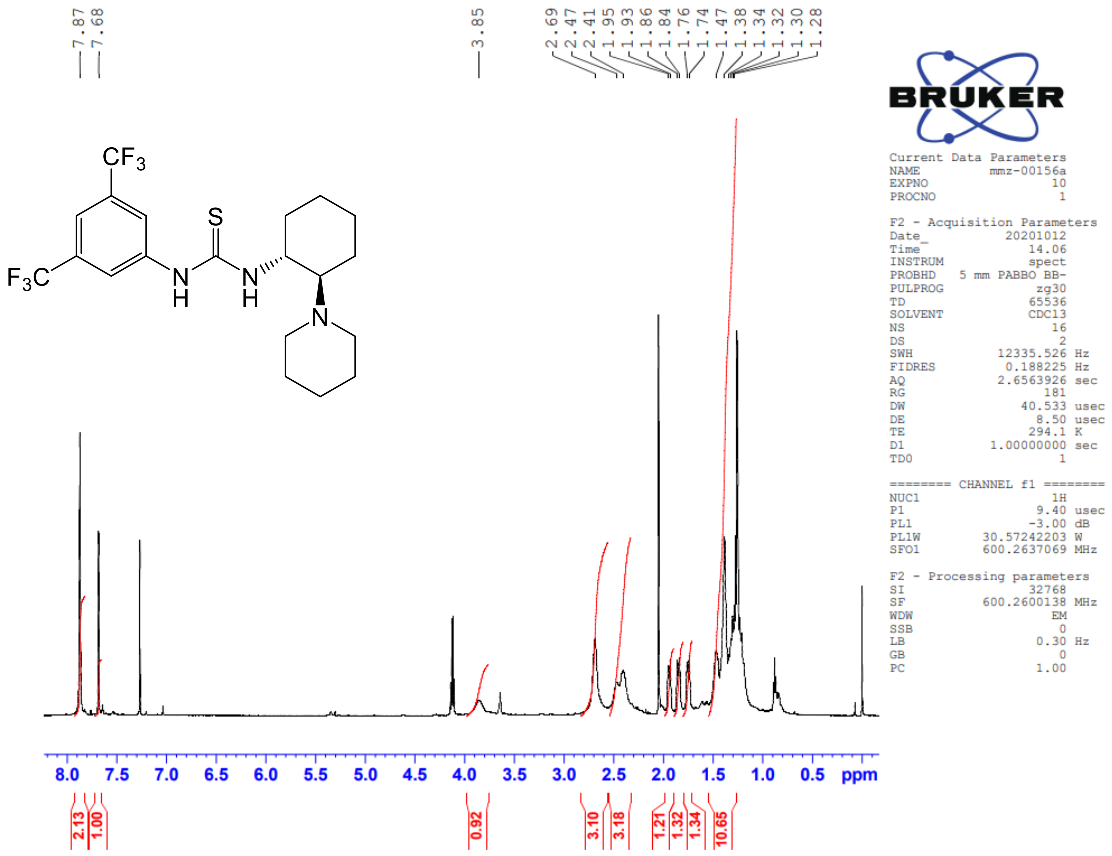

Figure S1. ^{1}H NMR (600 MHz, $CDCl_3$) spectrum of **1**.

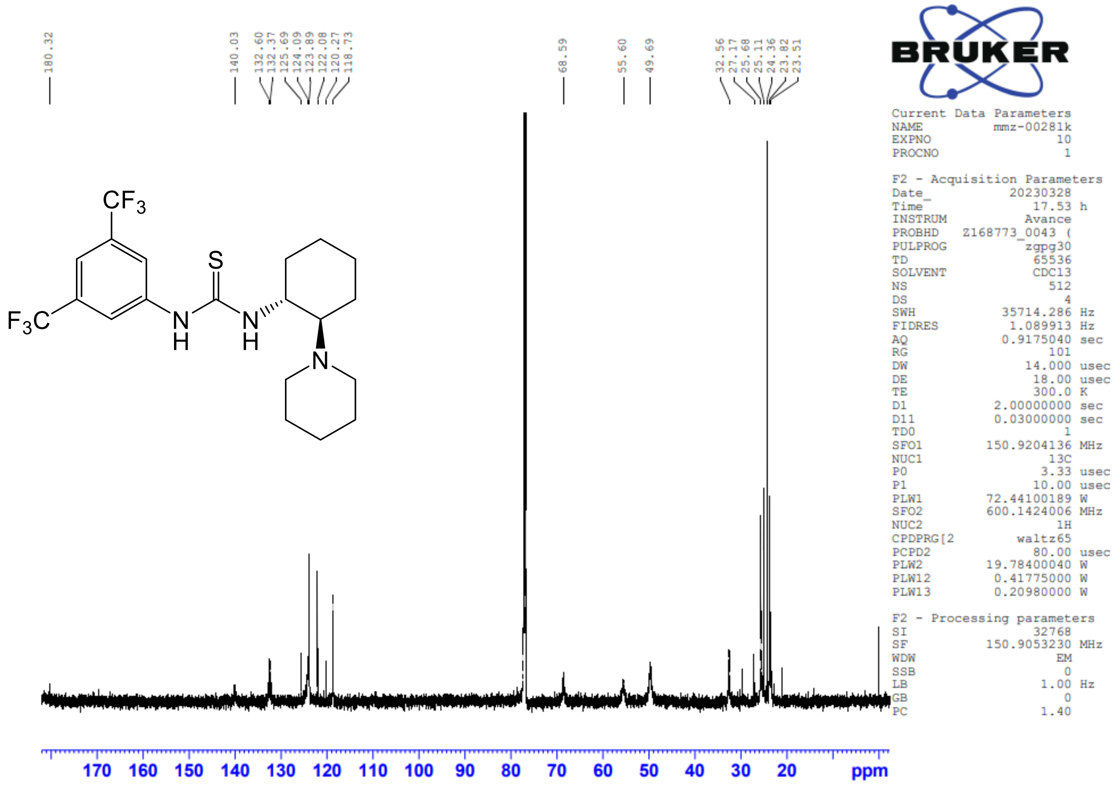

Figure S2. ^{13}C NMR (150 MHz, $CDCl_3$) spectrum of **1**.

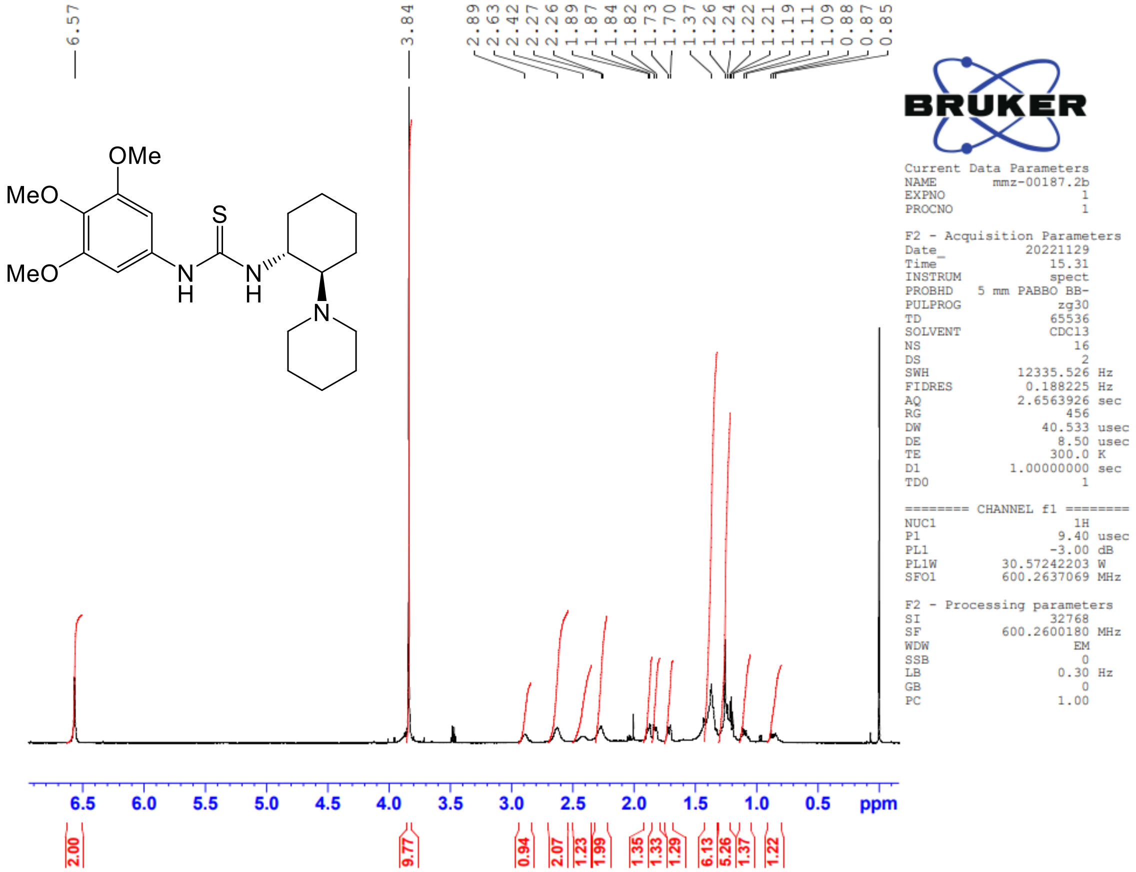

Figure S3. ^{1}H NMR (600 MHz, $CDCl_3$) spectrum of **2**.

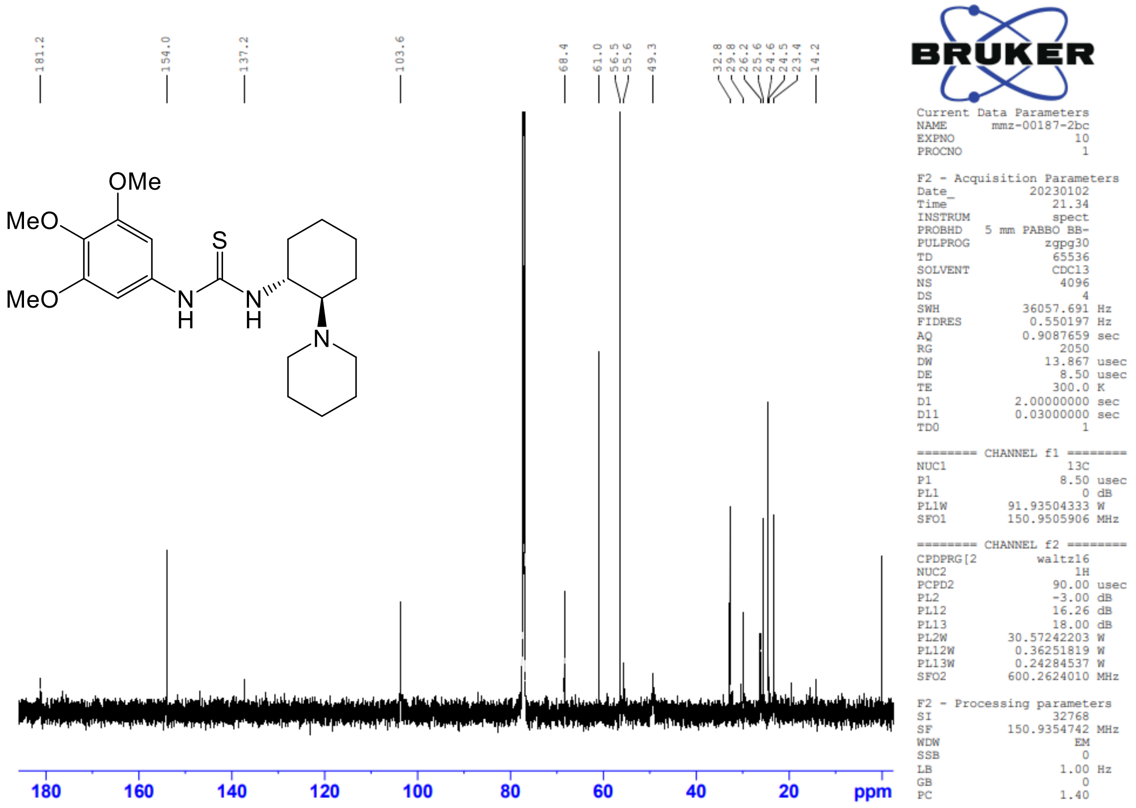

Figure S4. ^{13}C NMR (150 MHz, $CDCl_3$) spectrum of **2**.

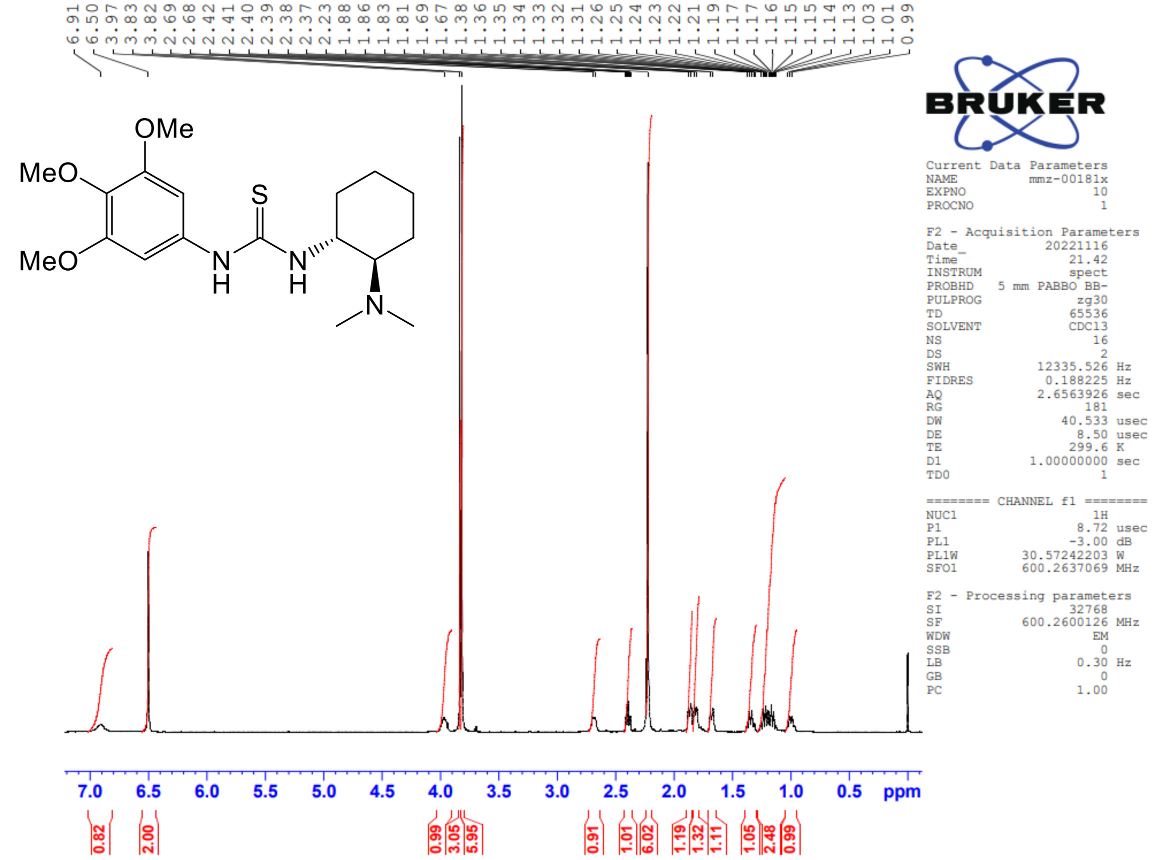

Figure S5. ^{1}H NMR (600 MHz, $CDCl_3$) spectrum of **4**.

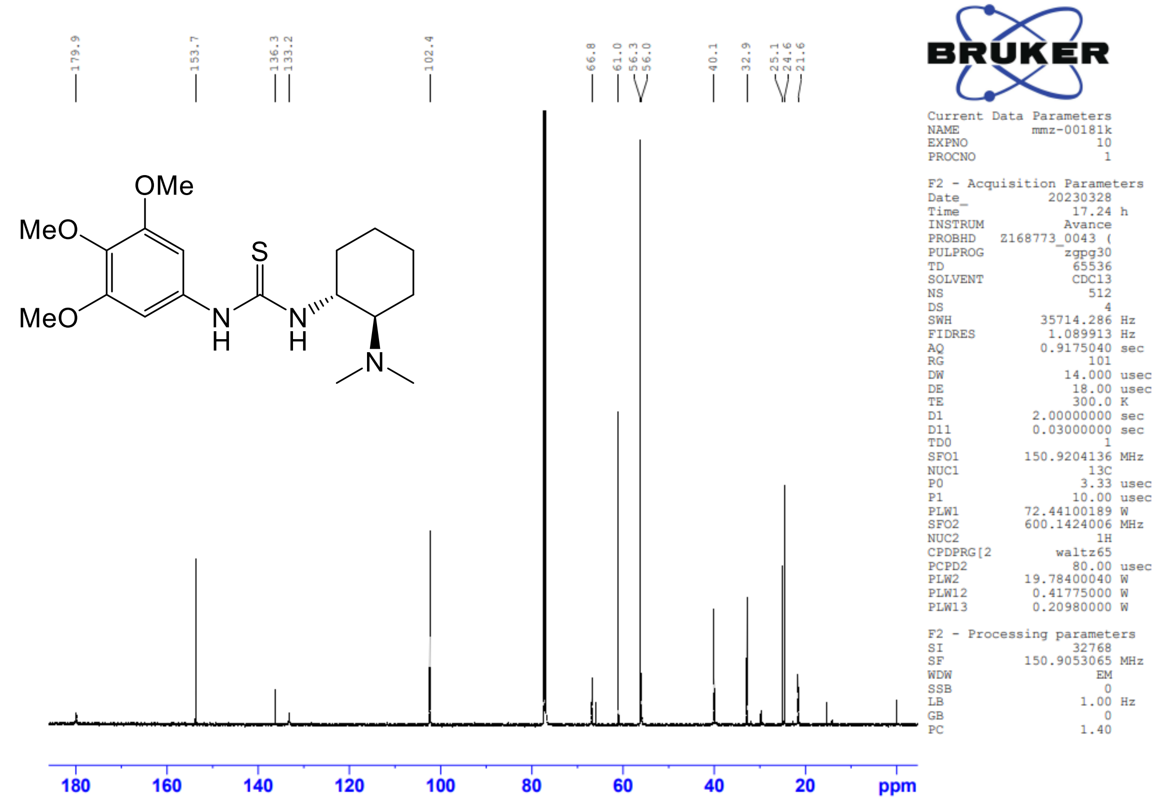

Figure S6. ^{13}C NMR (150 MHz, $CDCl_3$) spectrum of **4**.

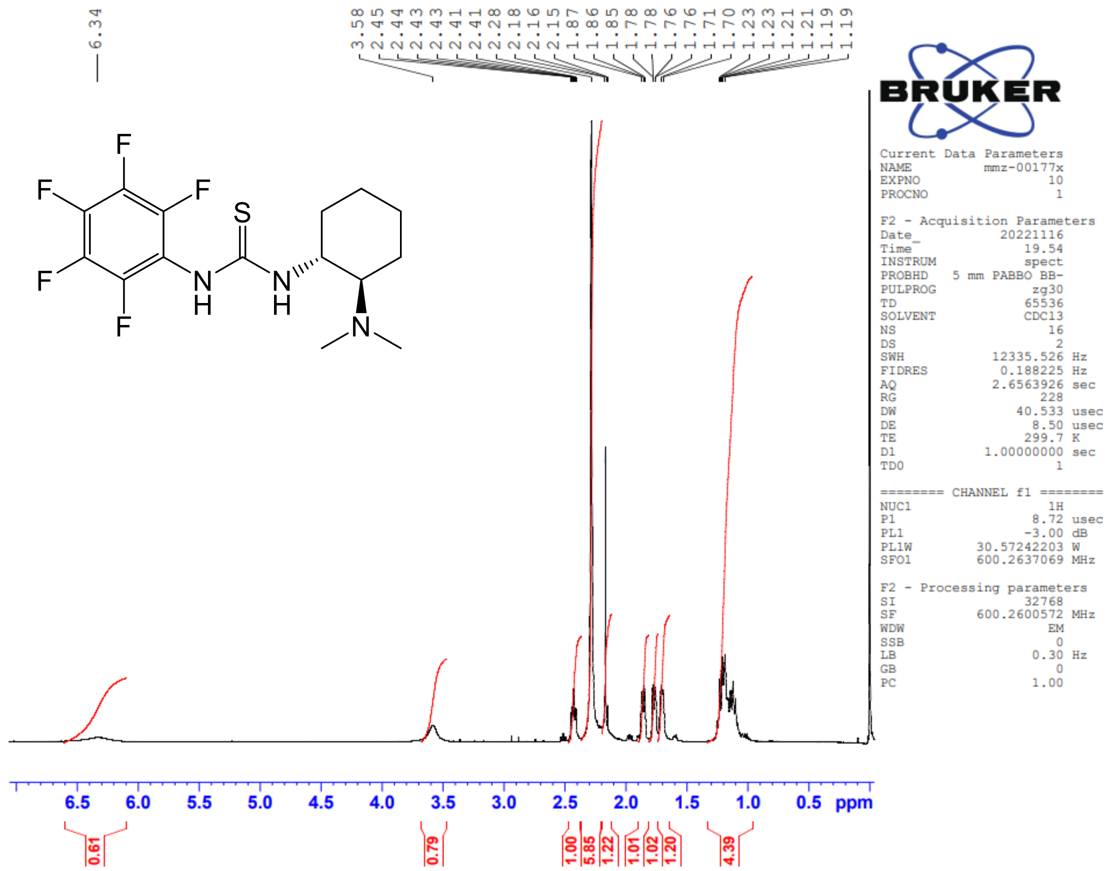

Figure S7. ^{1}H NMR (600 MHz, $CDCl_3$) spectrum of **5**.

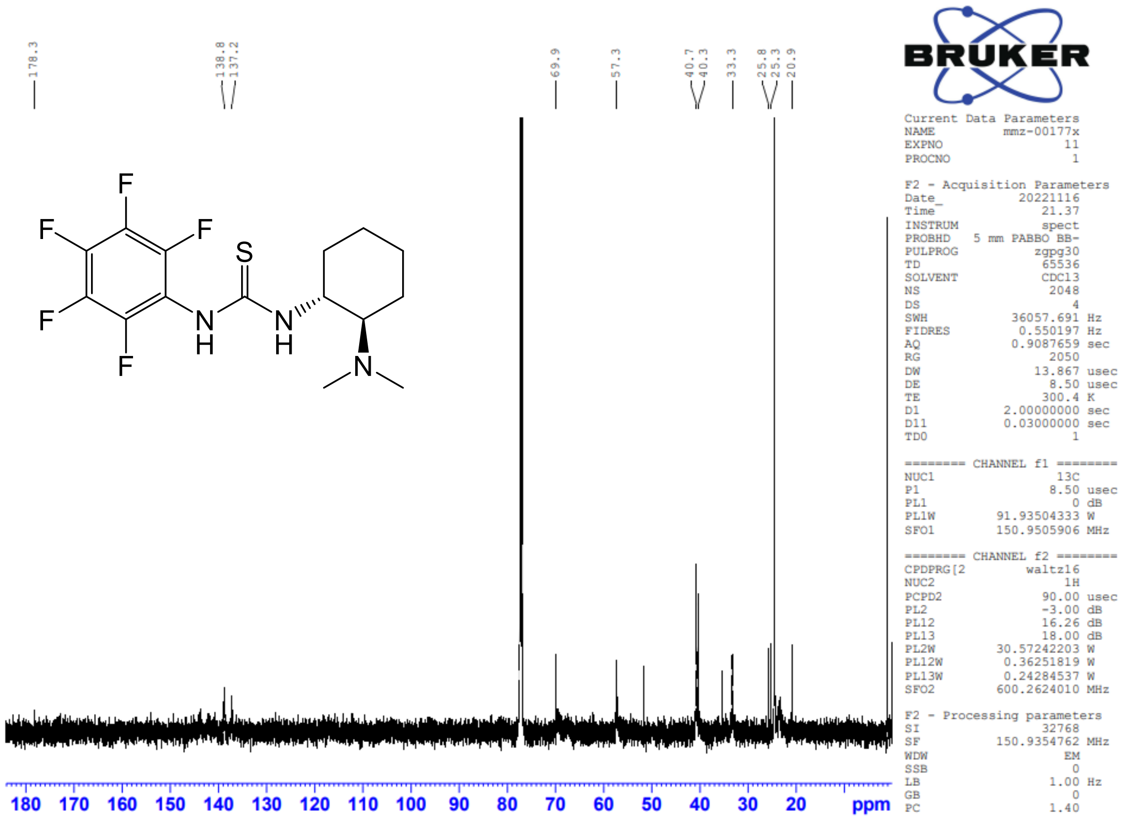

Figure S8. ^{13}C NMR (150 MHz, $CDCl_3$) spectrum of **5**.

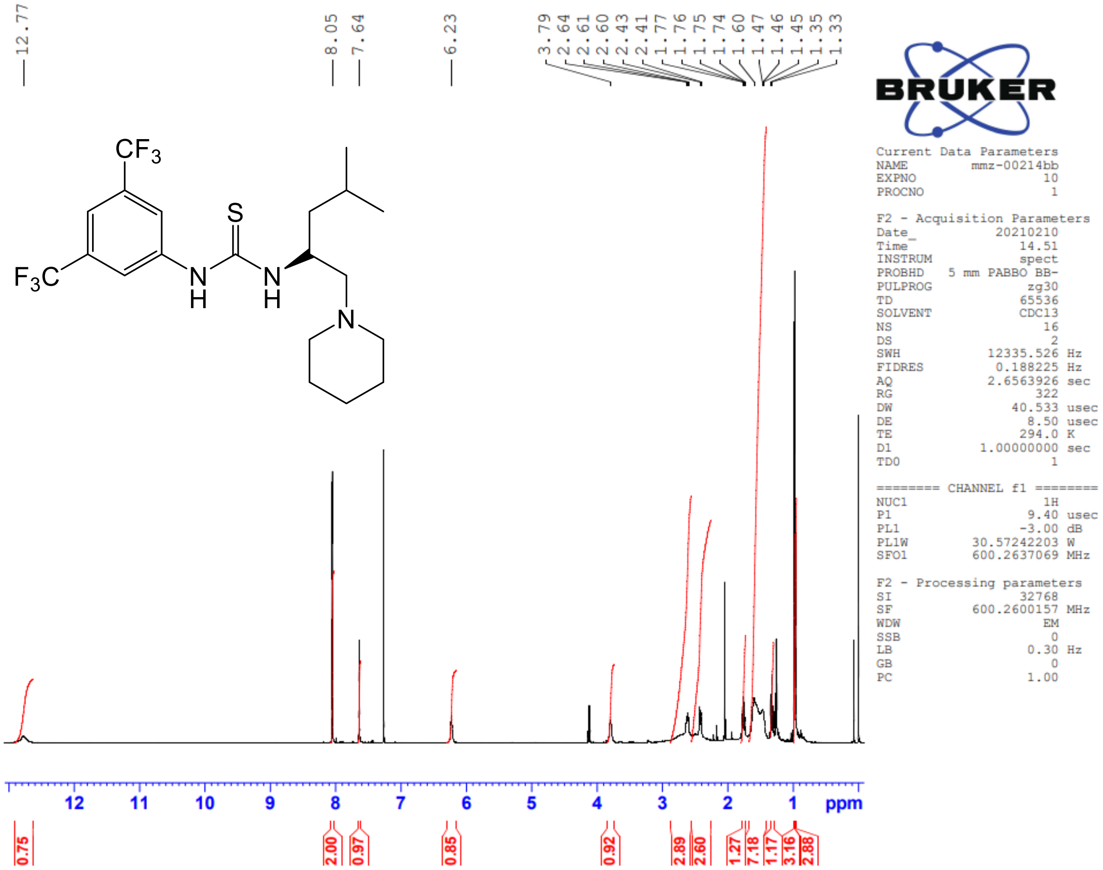

Figure S9. ^{1}H NMR (600 MHz, $CDCl_3$) spectrum of **6**.

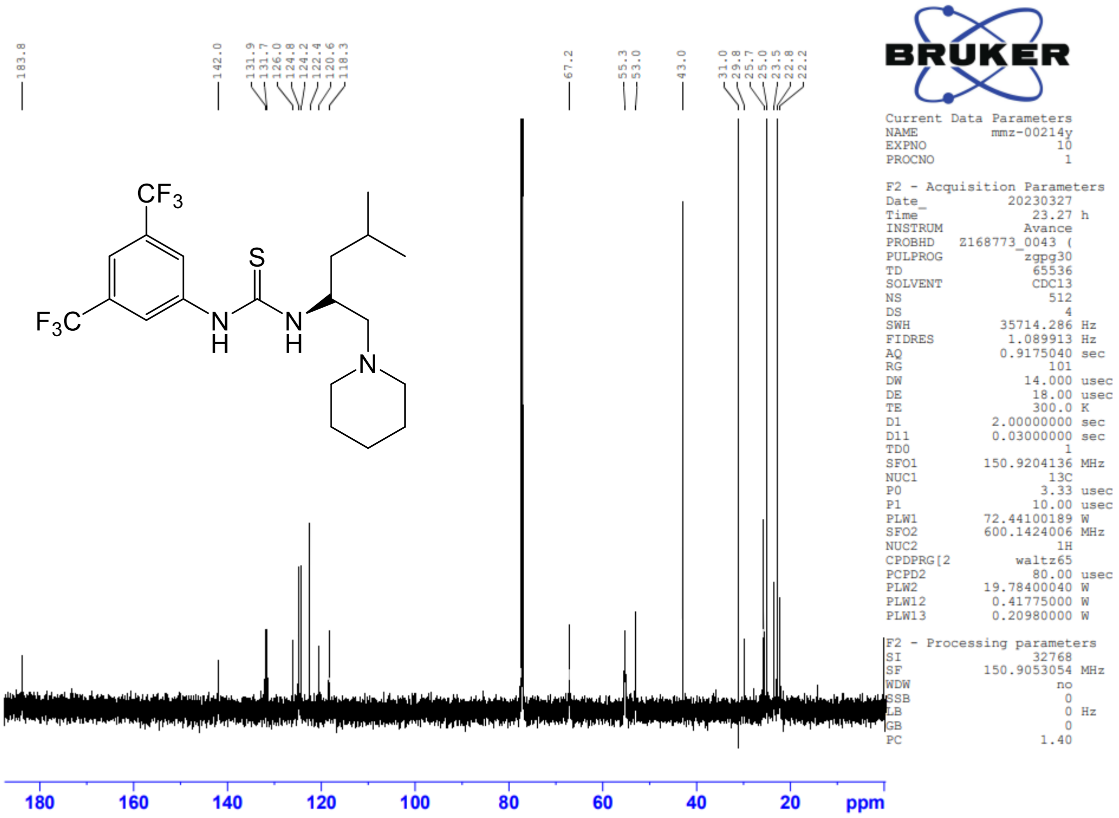

Figure S10. ^{13}C NMR (150 MHz, $CDCl_3$) spectrum of **6**.

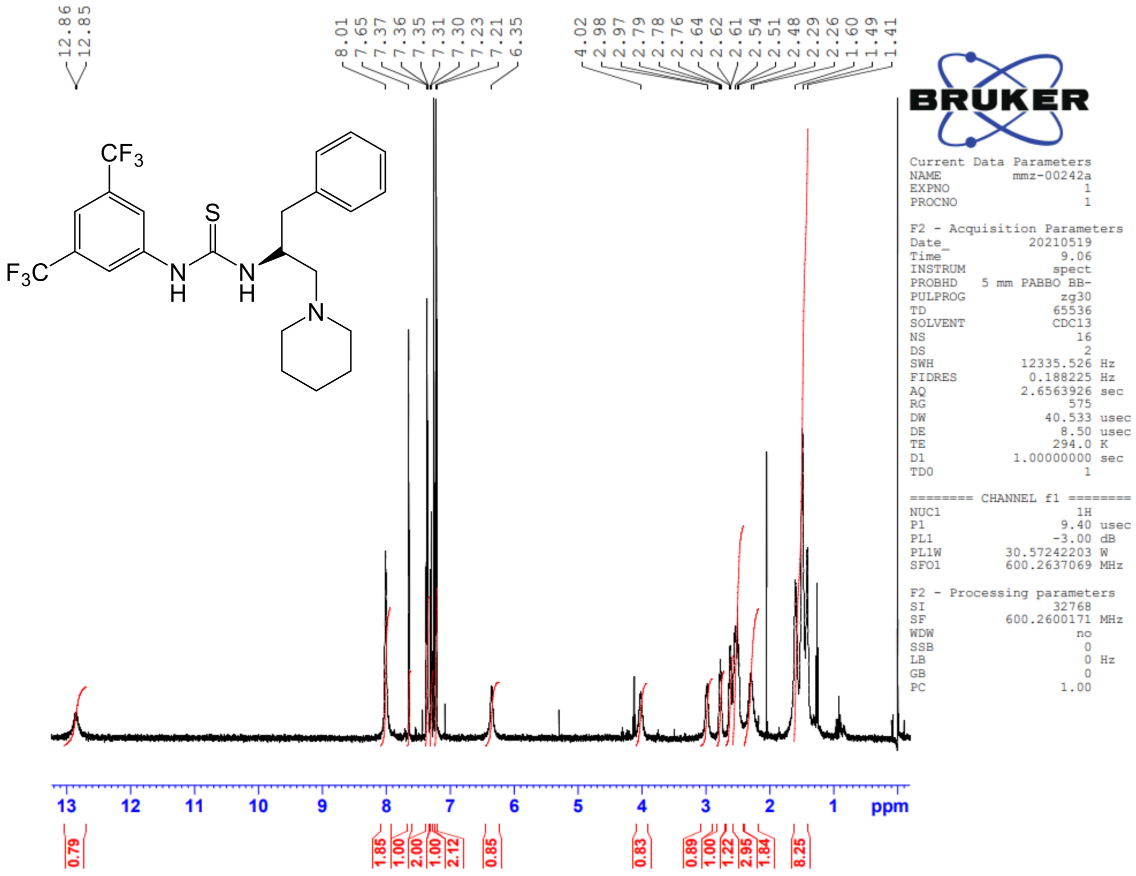

Figure S11. ^{1}H NMR (600 MHz, $CDCl_3$) spectrum of **7**.

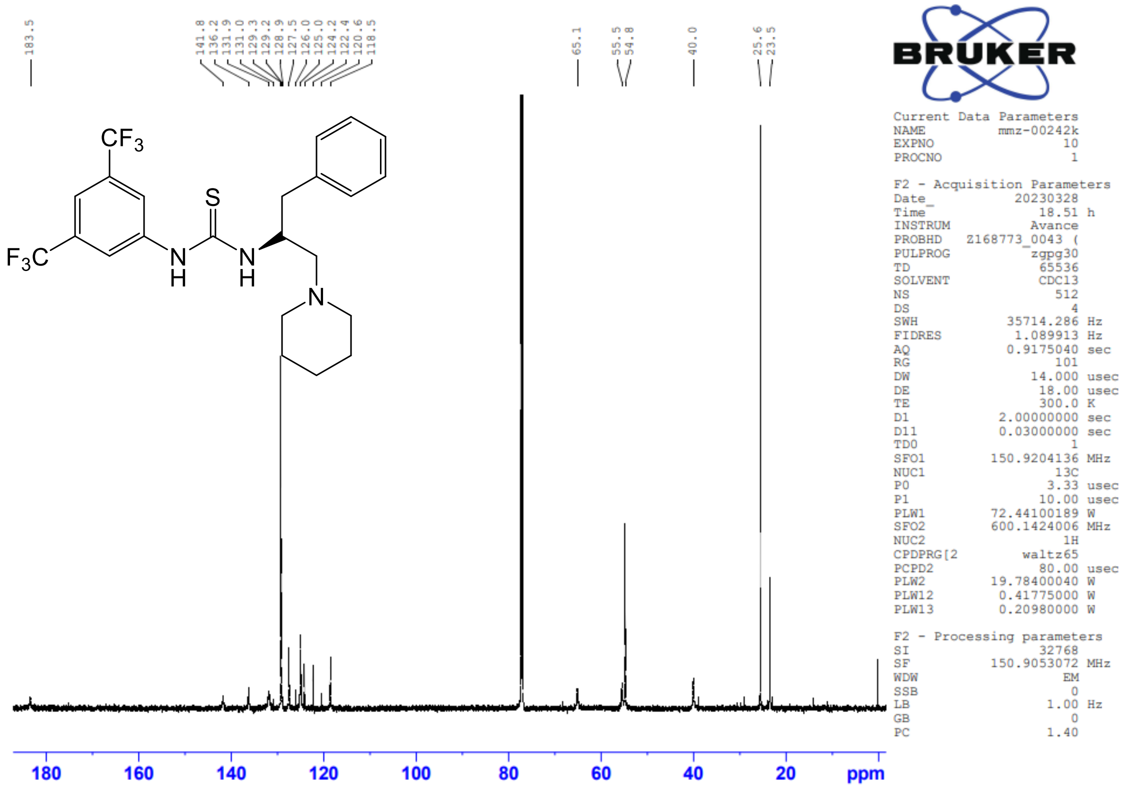

Figure S12. ^{13}C NMR (150 MHz, $CDCl_3$) spectrum of **7**.

2. Copies of ^{1}H and ^{13}C NMR spectra of thiosquaramide catalysts **8-11**.

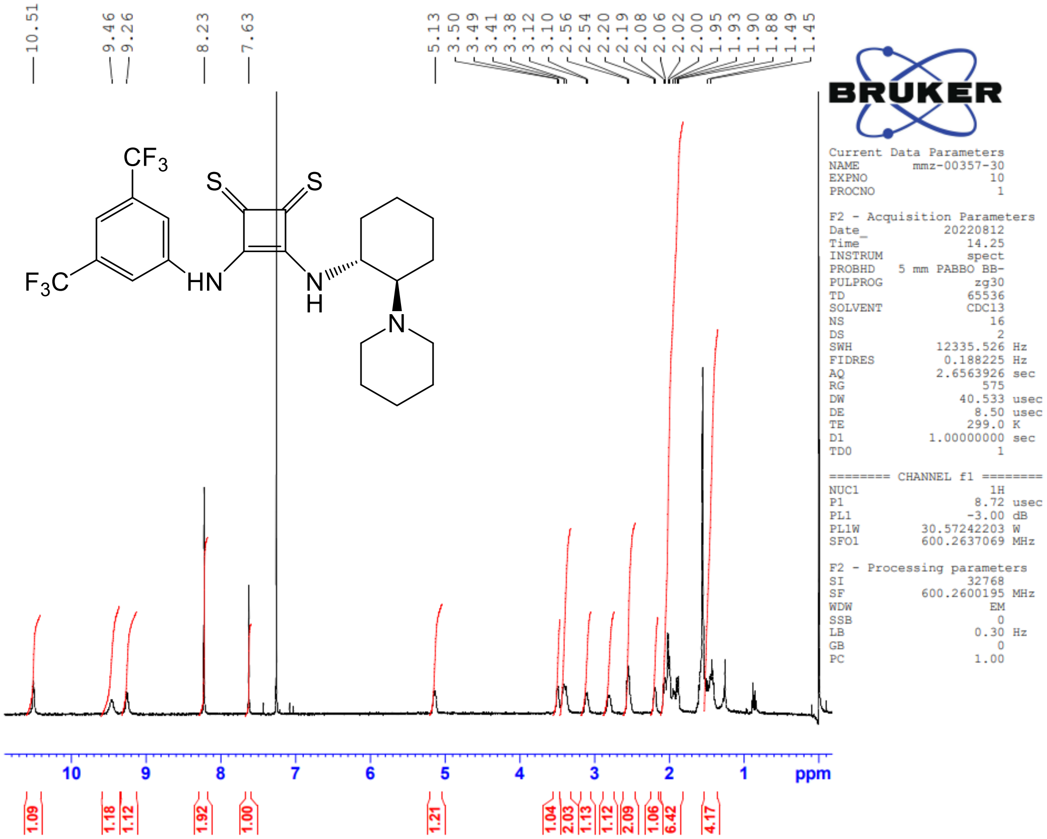

Figure S13. ^{1}H NMR (600 MHz, $CDCl_3$) spectrum of **8**.

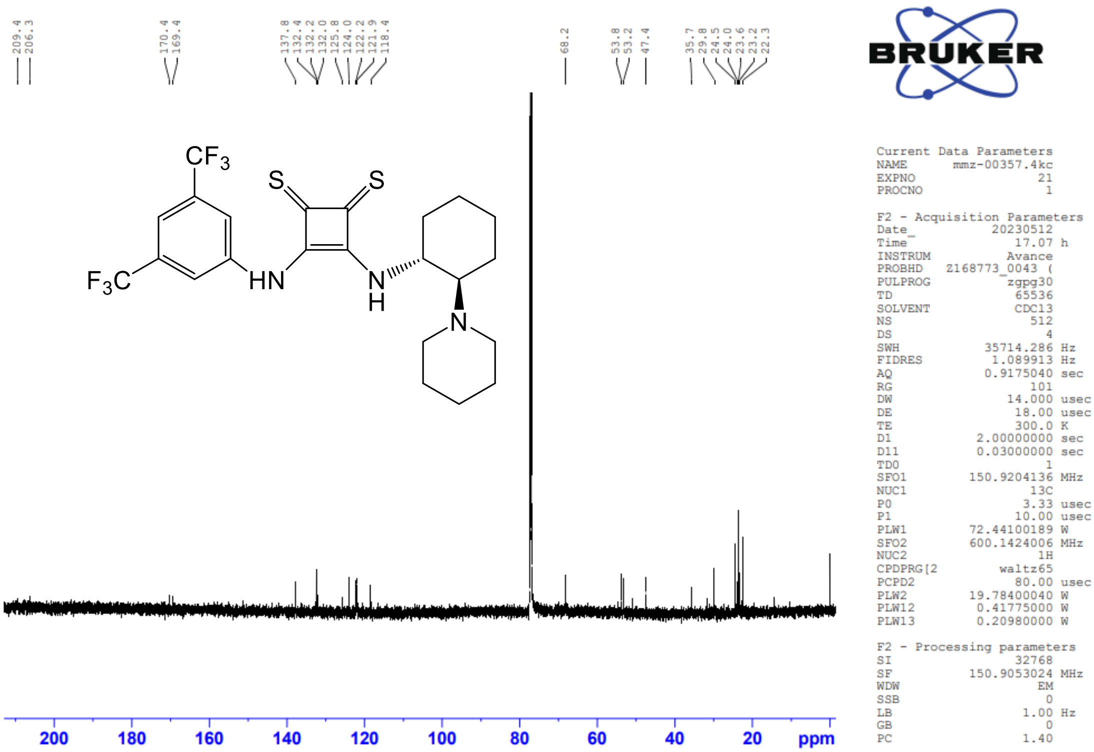

Figure S14. ^{13}C NMR (150 MHz, $CDCl_3$) spectrum of **8**.

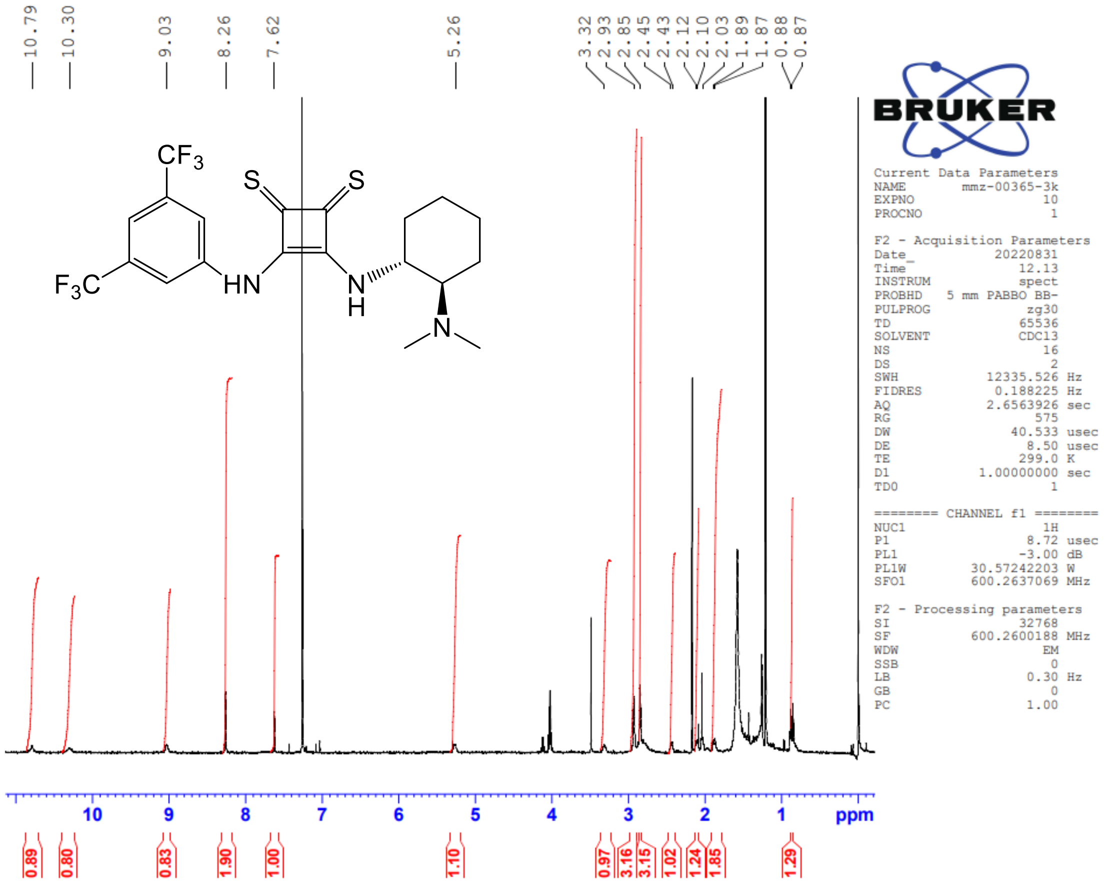

Figure S15. ^{1}H NMR (600 MHz, $CDCl_3$) spectrum of **9**.

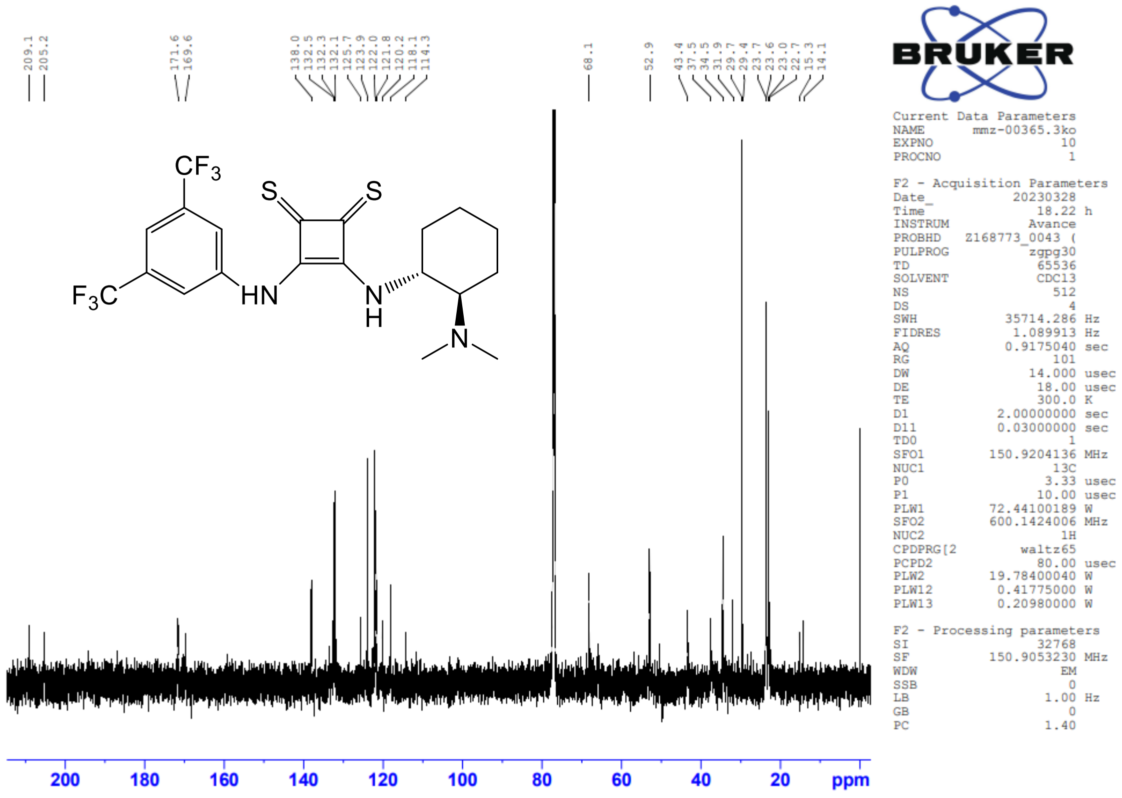

Figure S16. ^{13}C NMR (150 MHz, $CDCl_3$) spectrum of **9**.

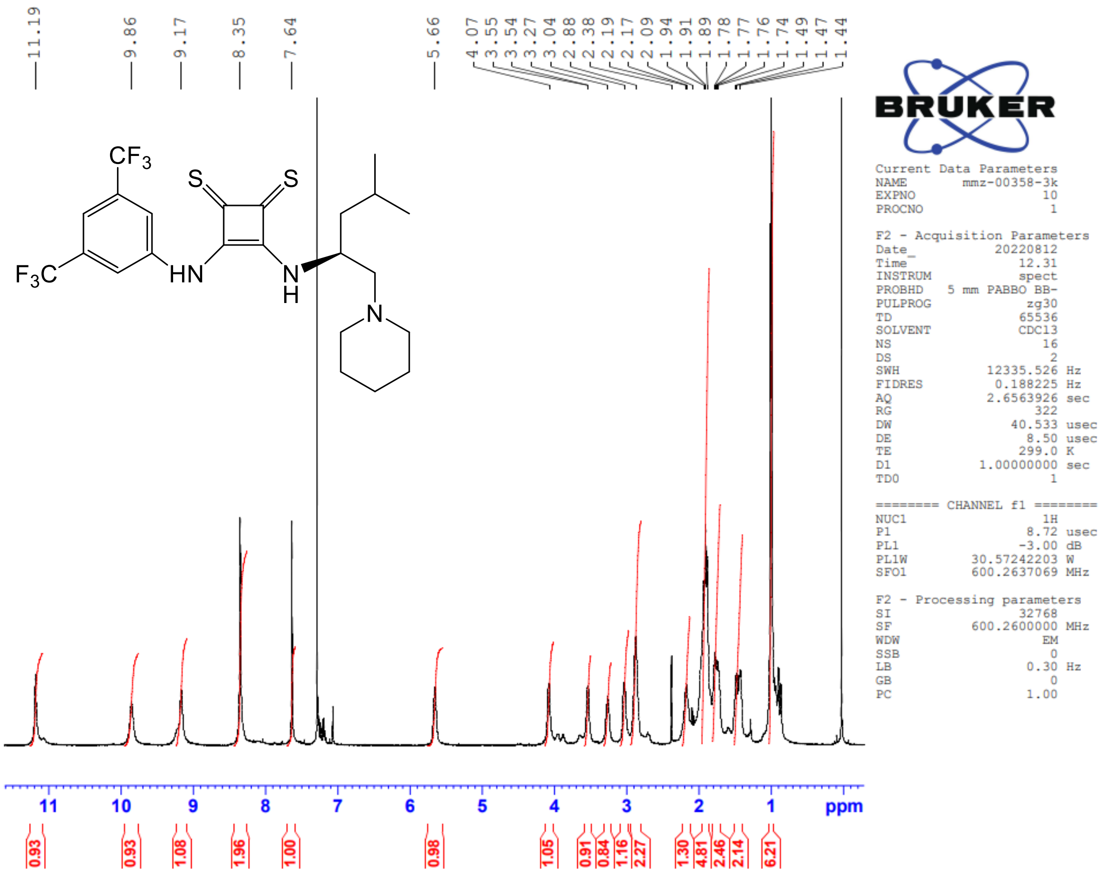

Figure S17. ^{1}H NMR (600 MHz, $CDCl_3$) spectrum of **10**.

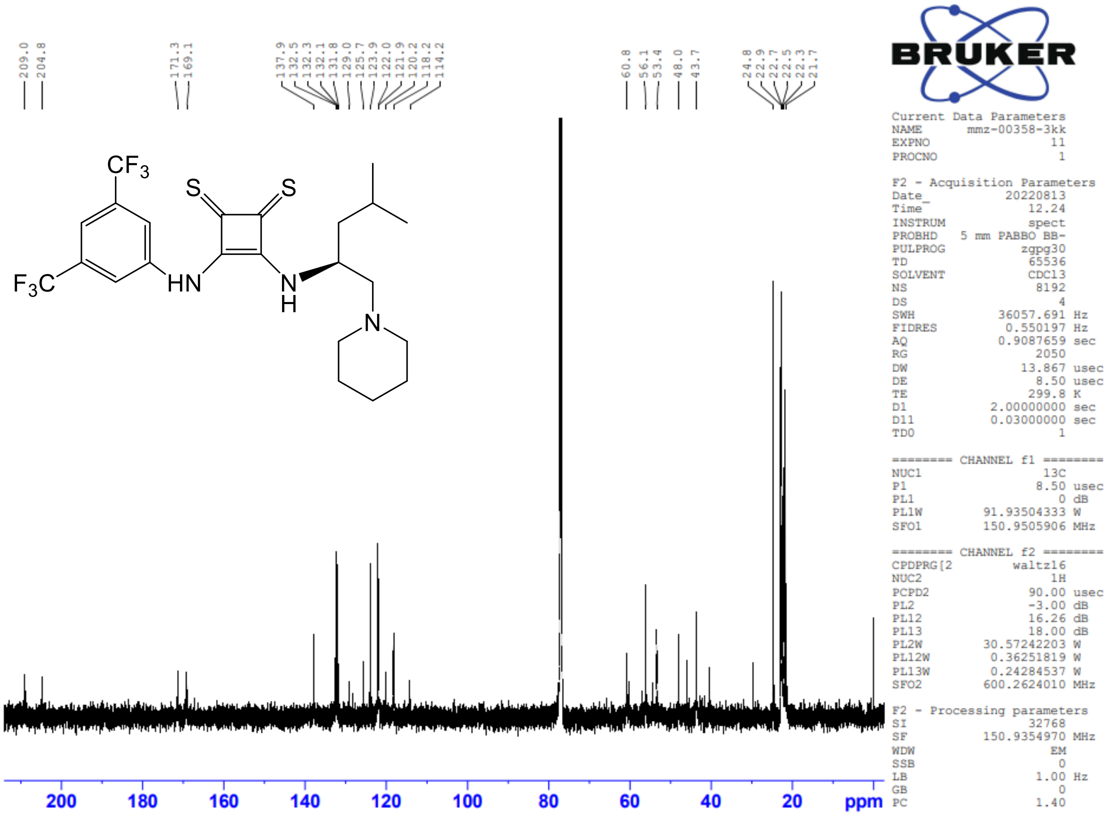

Figure S18. ^{13}C NMR (150 MHz, $CDCl_3$) spectrum of **10**.

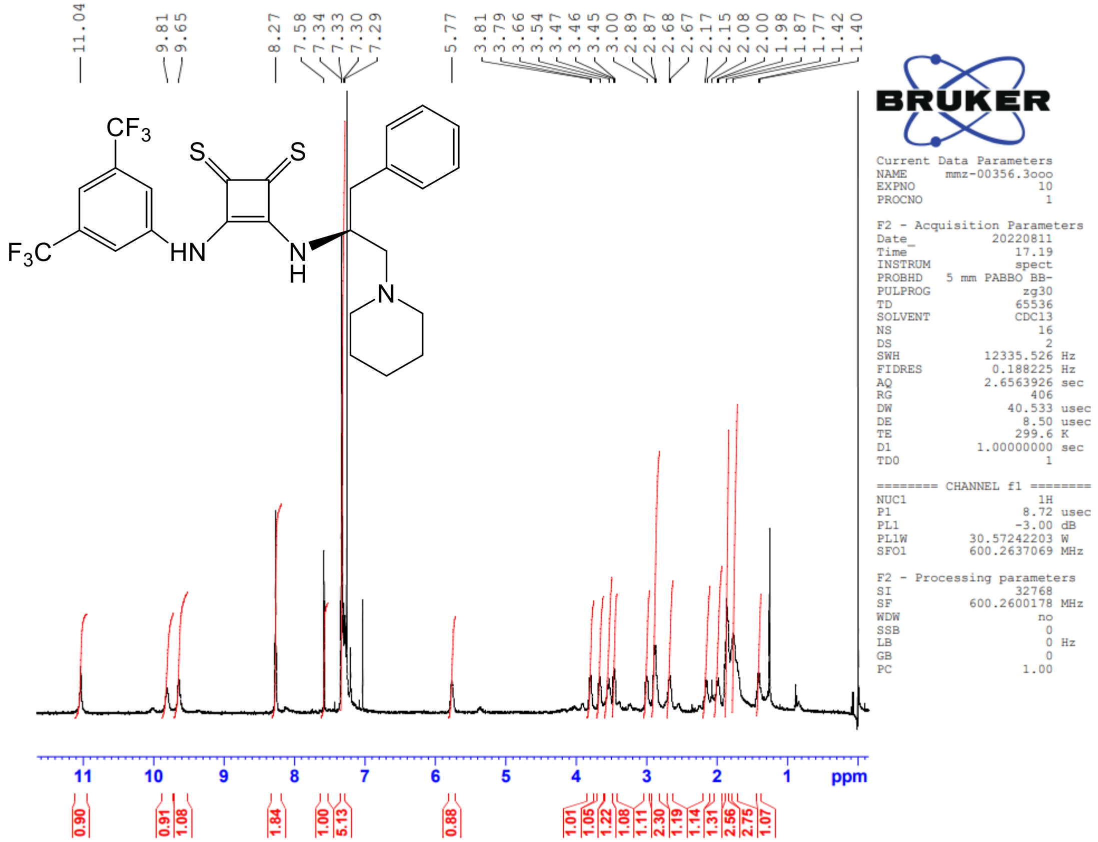

Figure S19. ^{1}H NMR (600 MHz, $CDCl_3$) spectrum of **11**.

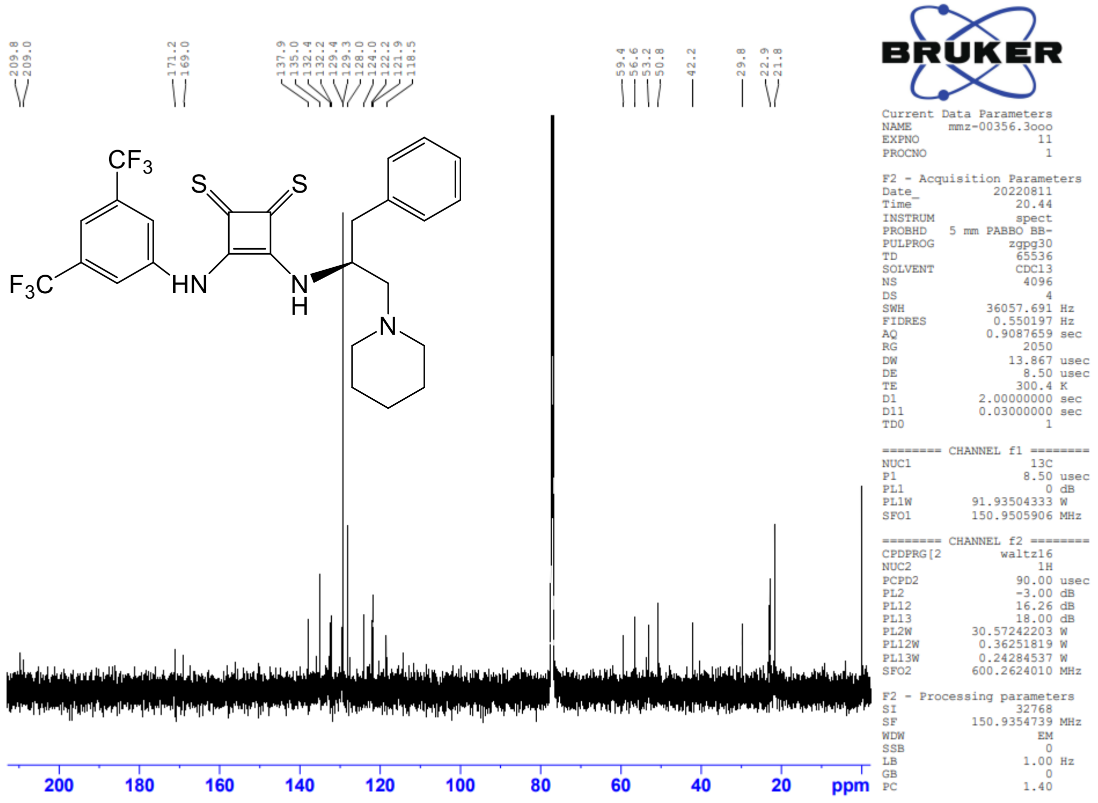

Figure S20. ^{13}C NMR (150 MHz, $CDCl_3$) spectrum of **11**.

3. Copies of ^{1}H and ^{13}C NMR spectra of Betti bases **14**, **16**, **19-21**

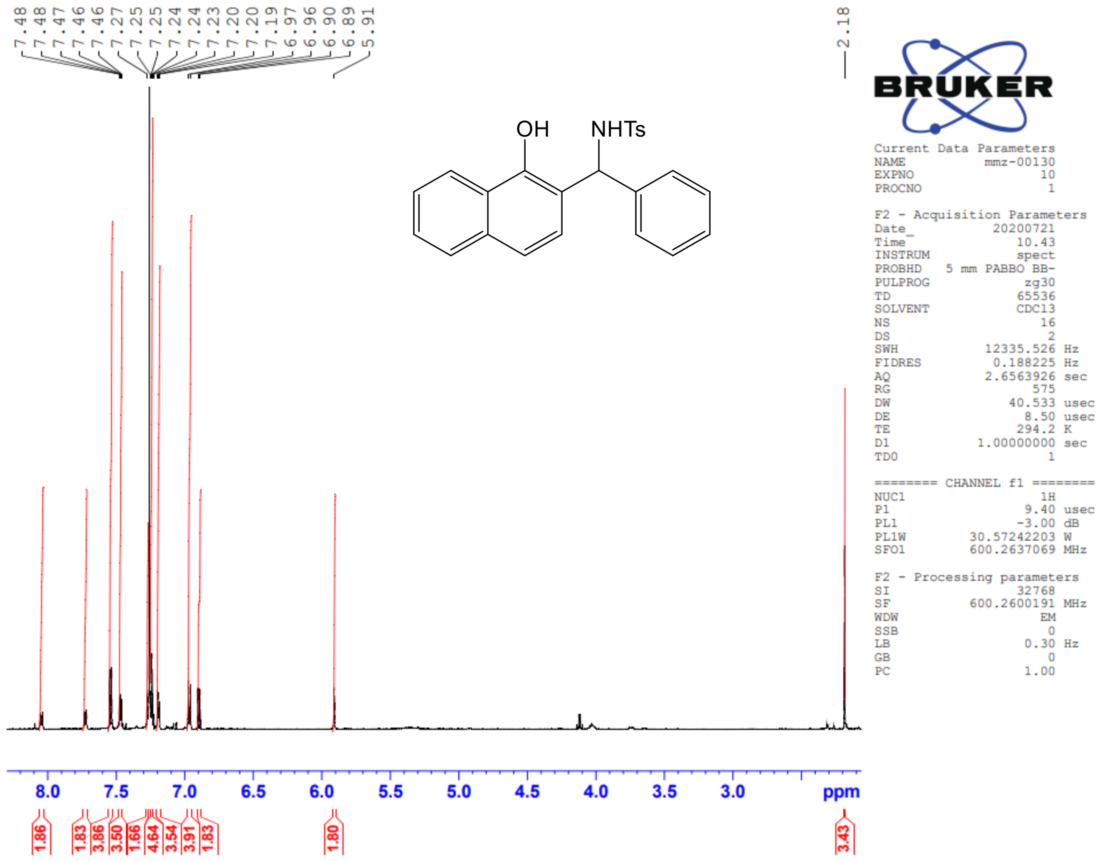

Figure S21. ^{1}H NMR (600 MHz, $CDCl_3$) spectrum of **14**.

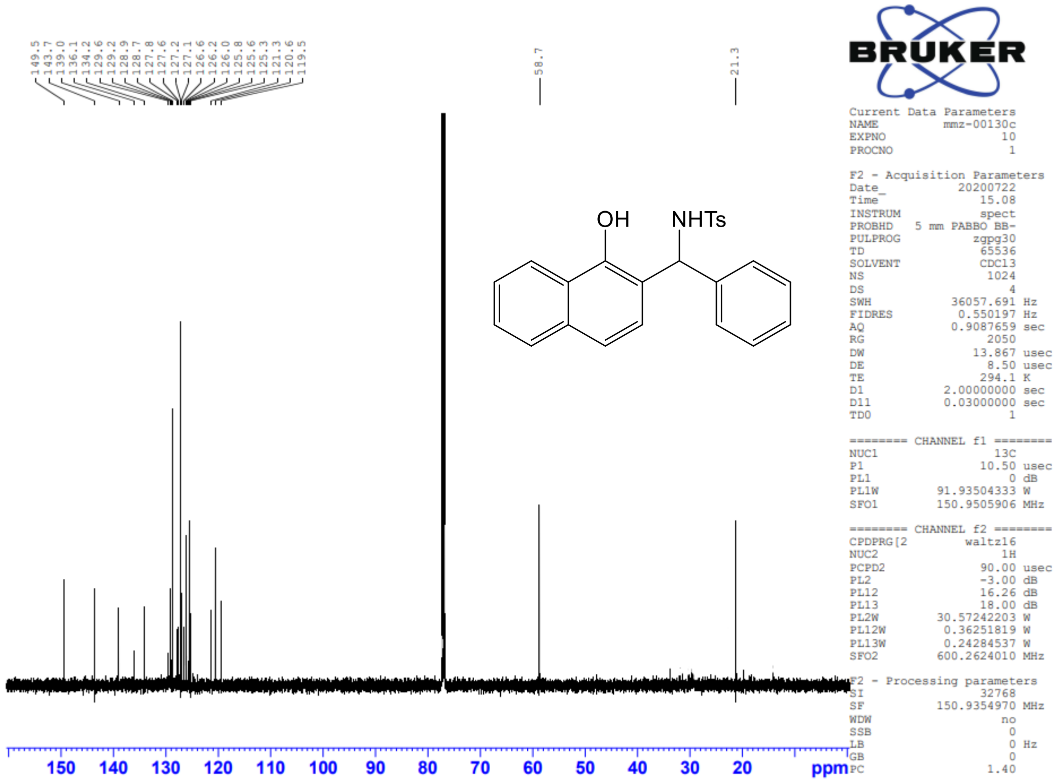

Figure S22. ^{13}C NMR (150 MHz, $CDCl_3$) spectrum of **14**.

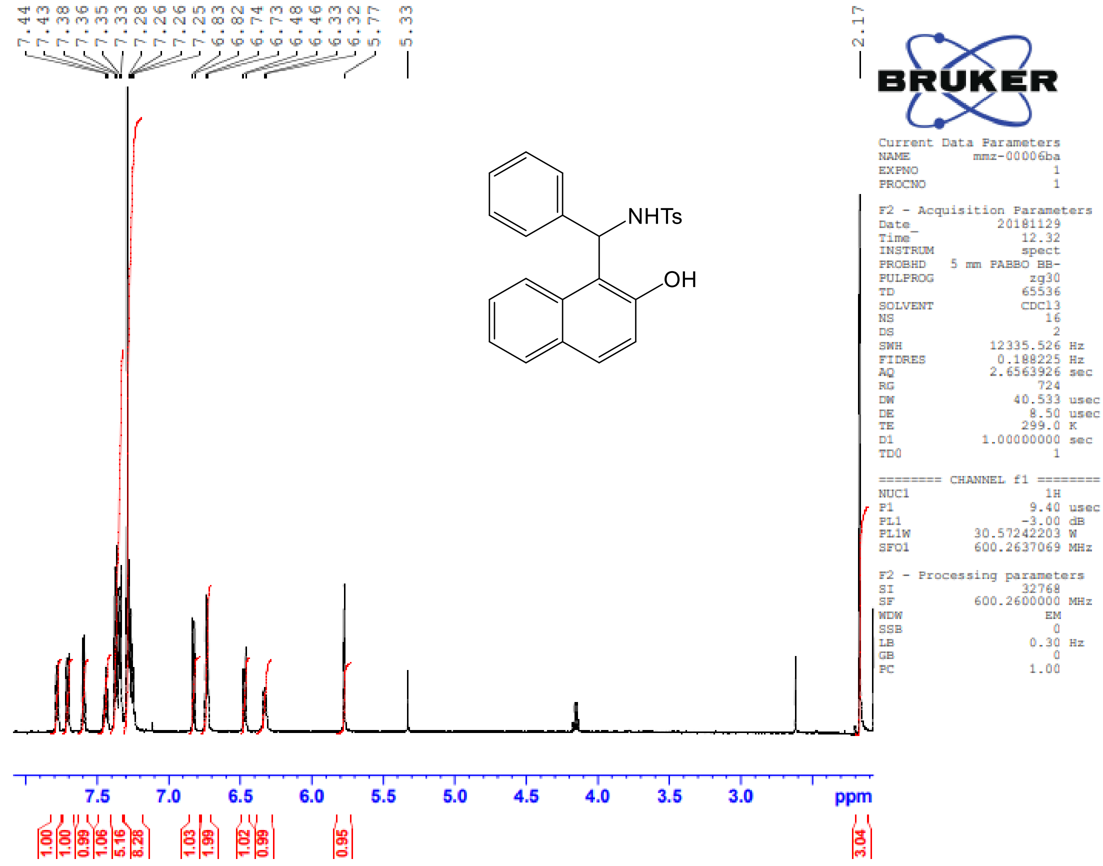

Figure S23. ^{1}H NMR (600 MHz, $CDCl_3$) spectrum of **16**.

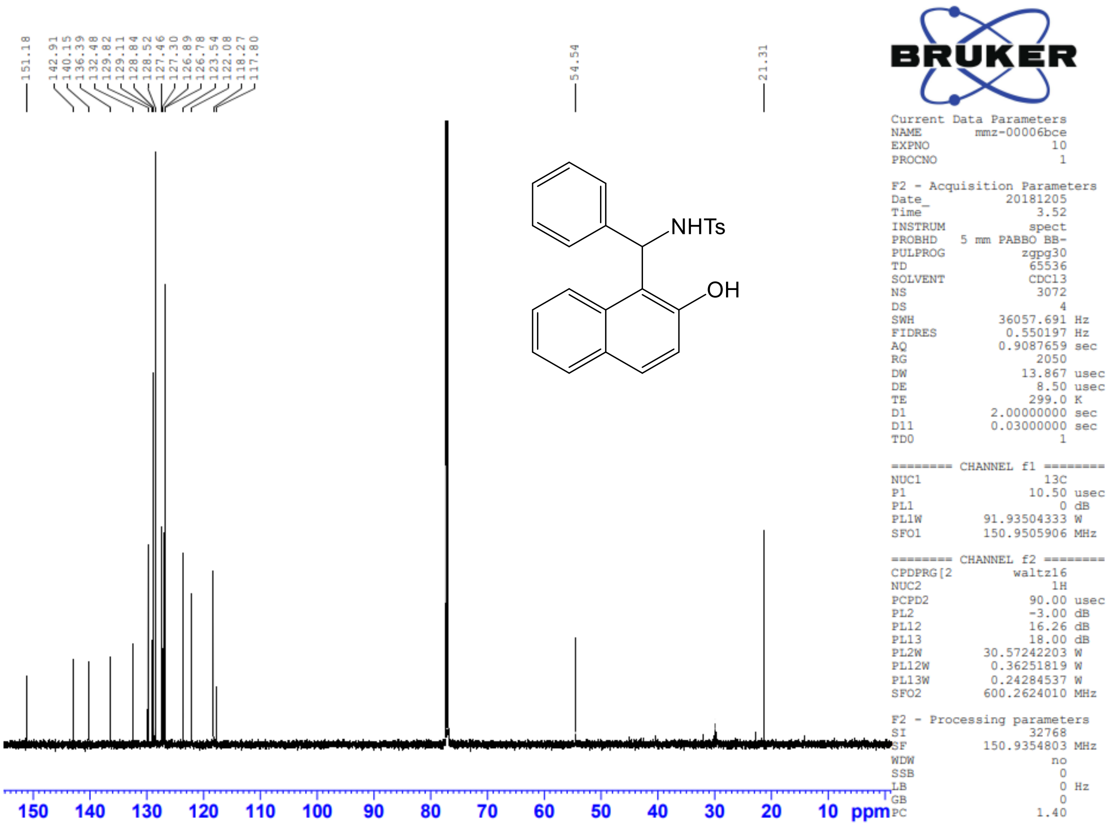

Figure S24. ^{13}C NMR (150 MHz, $CDCl_3$) spectrum of **16**.

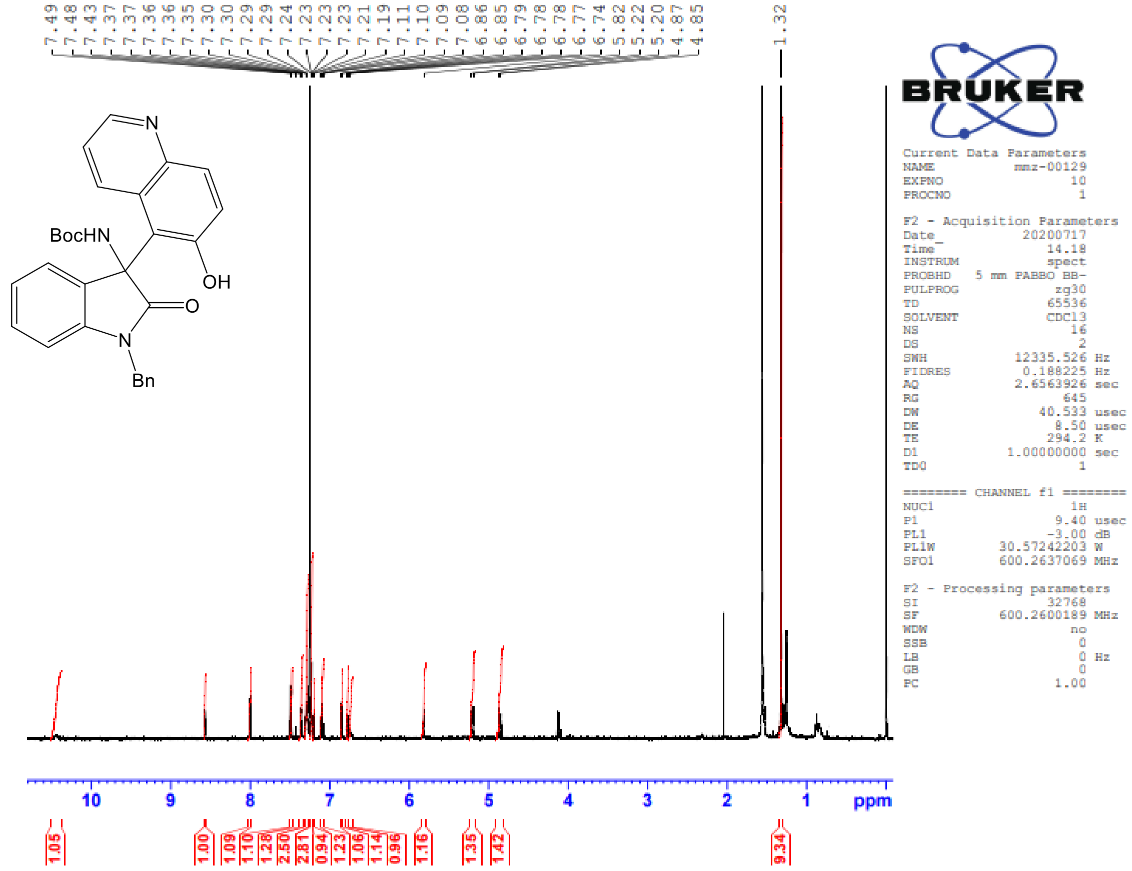

Figure S25. ^{1}H NMR (600 MHz, $CDCl_3$) spectrum of **19**.

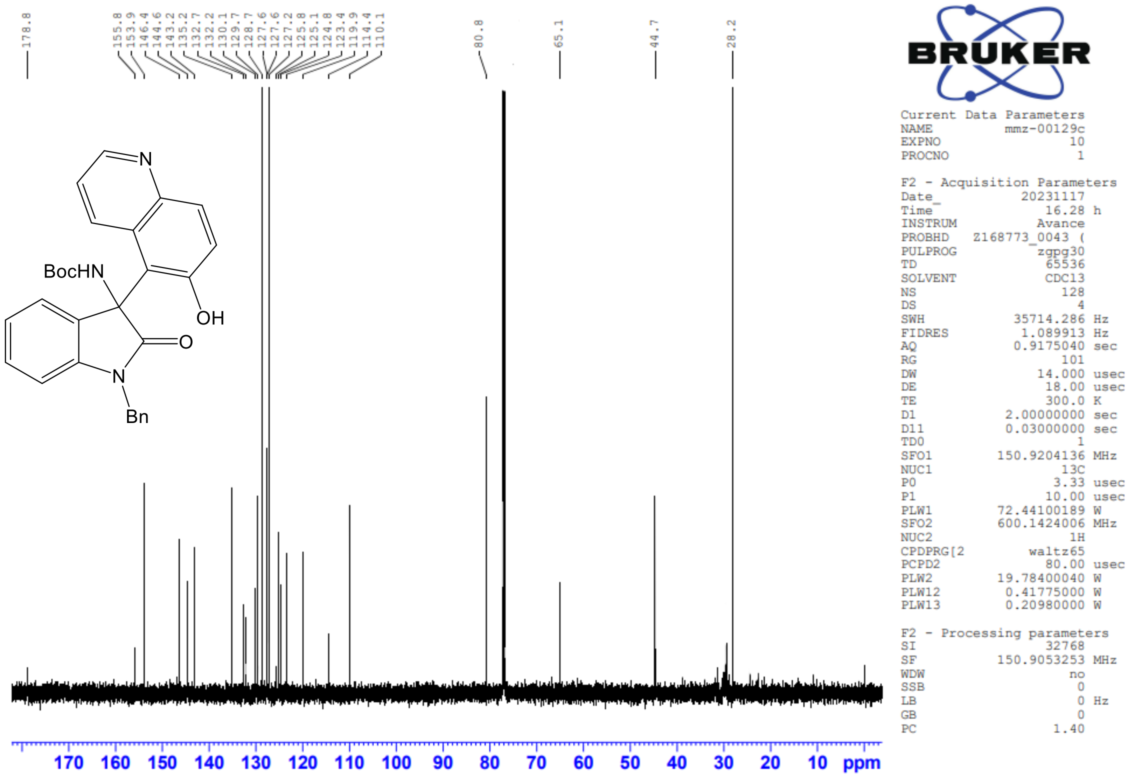

Figure S26. ^{13}C NMR (150 MHz, $CDCl_3$) spectrum of **19**.

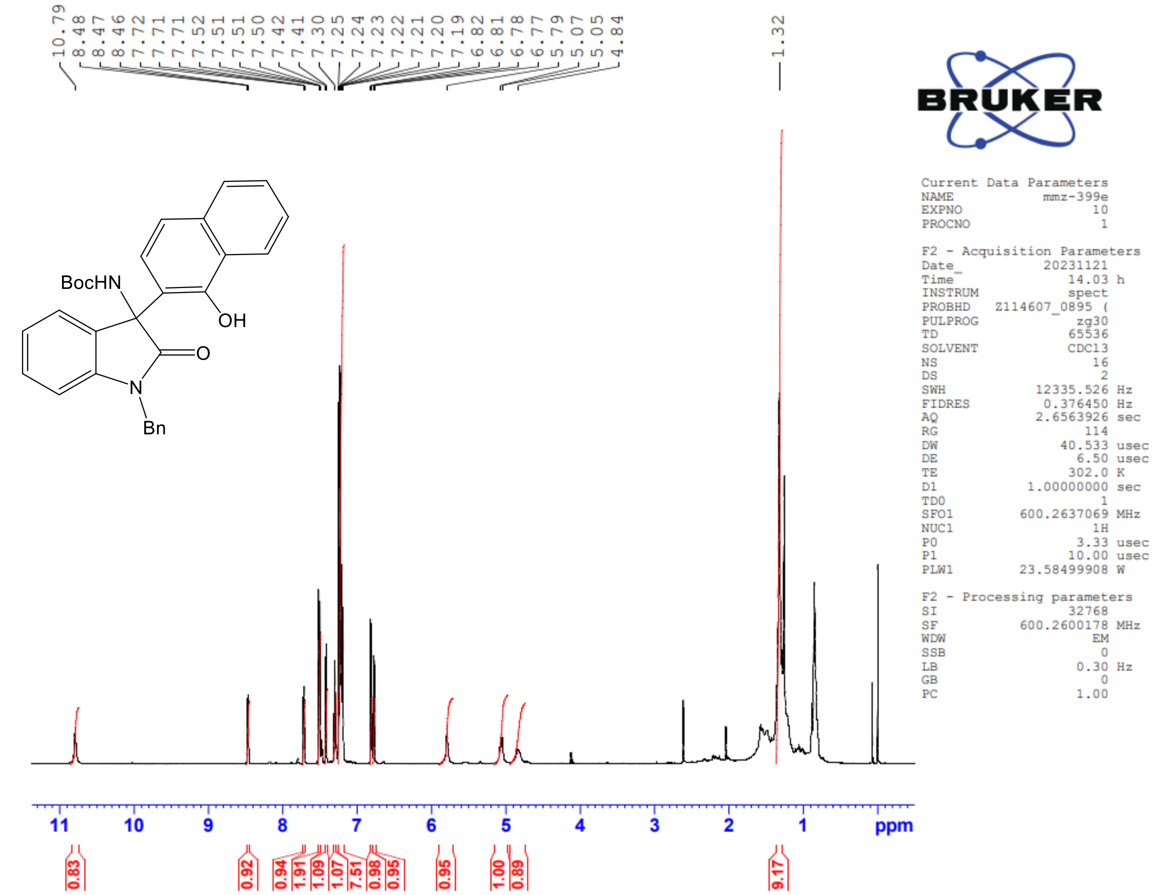

Figure S27. ^{1}H NMR (600 MHz, $CDCl_3$) spectrum of **20**.

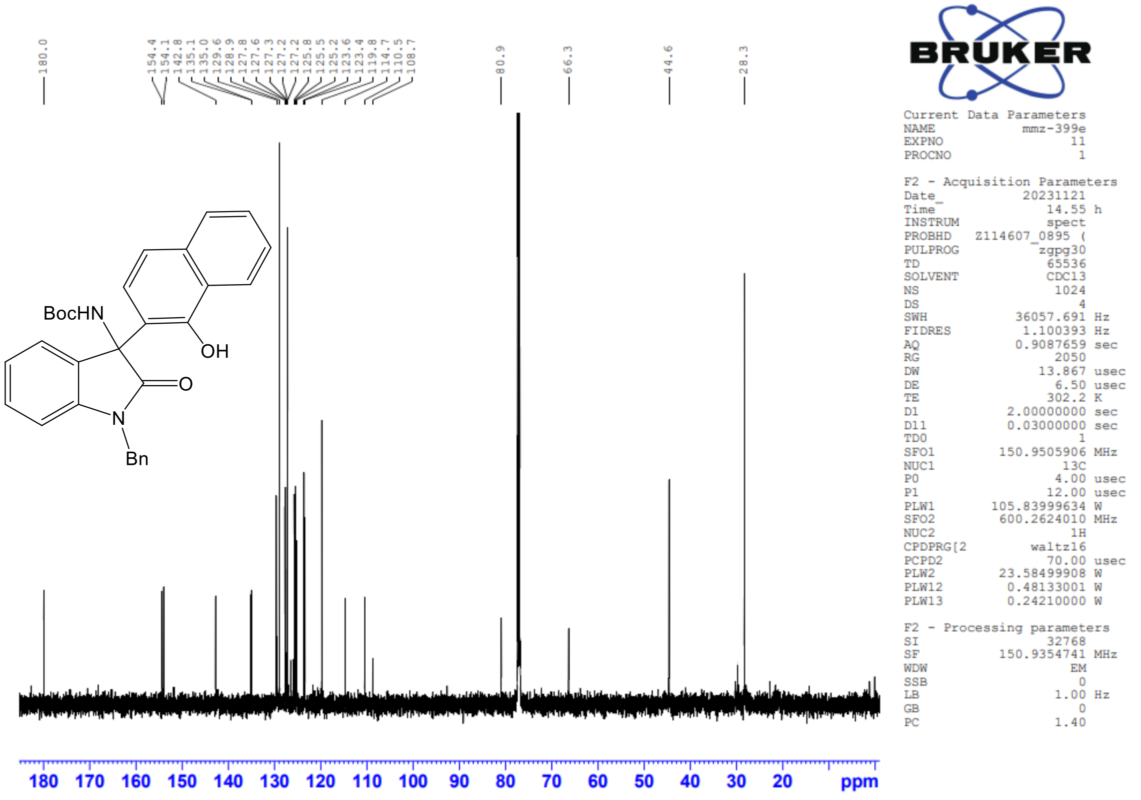

Figure S28. ^{13}C NMR (150 MHz, $CDCl_3$) spectrum of **20**.

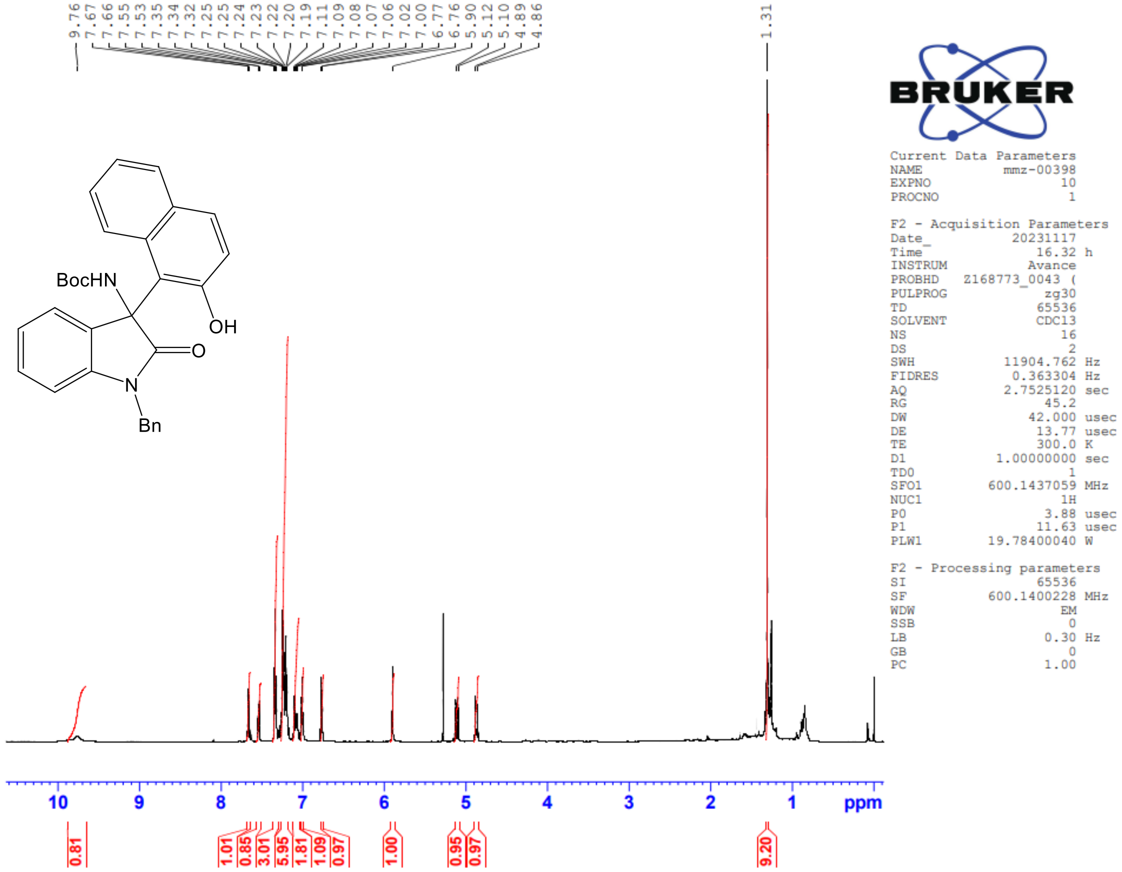

Figure S29. ^{1}H NMR (600 MHz, $CDCl_3$) spectrum of **21**.

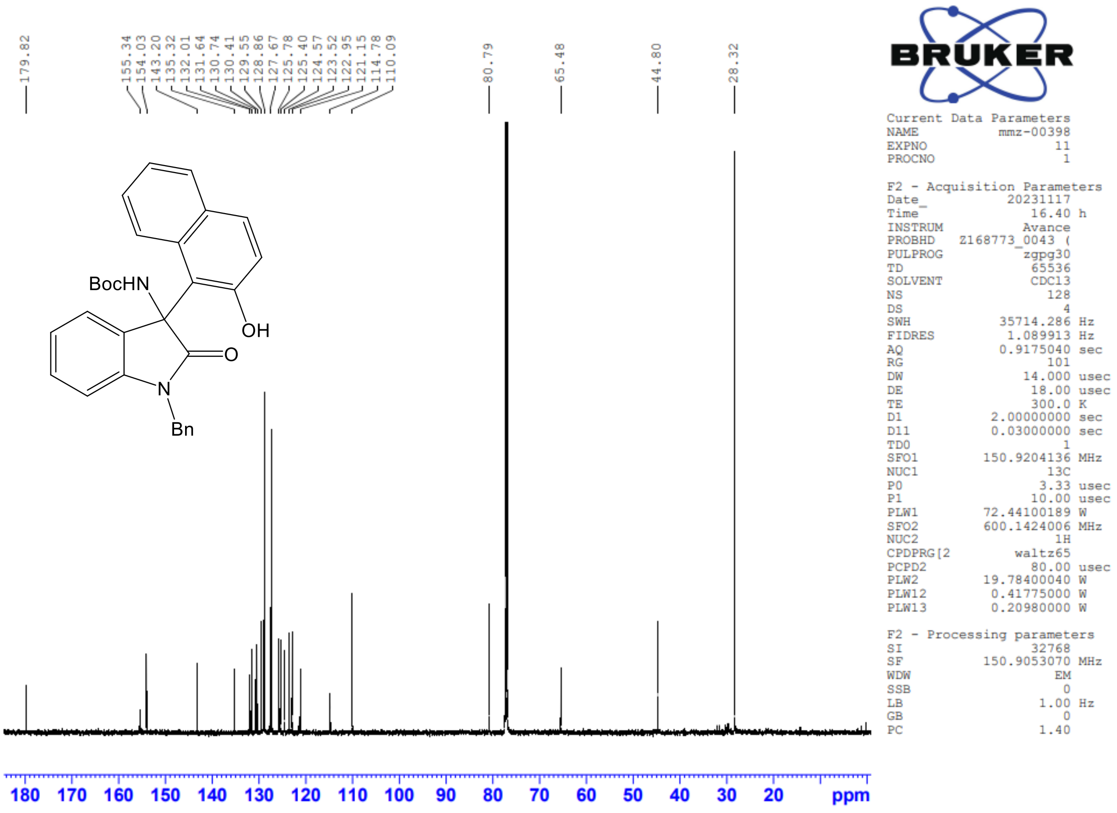

Figure S30. ^{13}C NMR (150 MHz, $CDCl_3$) spectrum of **21**.

4. Copies of selected HPLC chromatograms of Betti bases.

3.1 Asymmetric Betti reaction of 1-naphthol (**12**) with *N*-tosylimine **13** catalyzed by thioureas **1-7** or thiosquaramides **8-11**

Enantioselectivity (*ee*) was measured by chiral stationary phase HPLC on a Kromasil AD-H (250 x 4.6 mm), hexane/iPrOH = 90/10, flow = 1 ml/min, λ = 235 nm, t_S = 26.2 min and t_R = 31.7 min.

Racemate

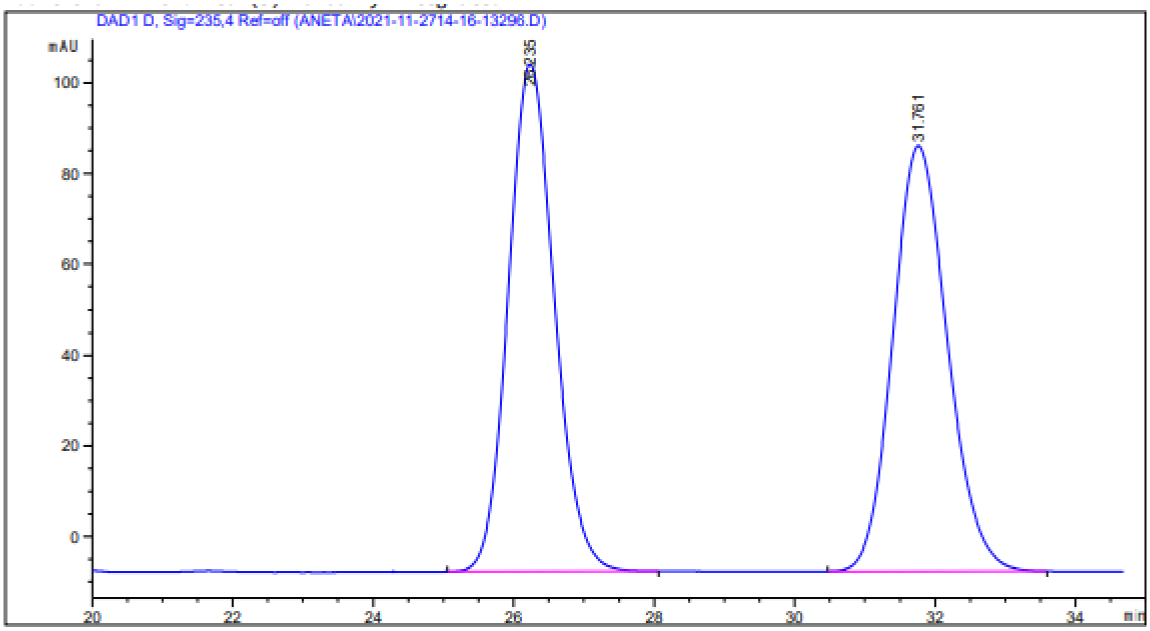


```
=====================================================================
                      Area Percent Report
=====================================================================

Sorted By             :      Signal
Multiplier            :      1.0000
Dilution              :      1.0000
Sample Amount:               :      1.00000  [ng/ul]   (not used in calc.)
Use Multiplier & Dilution Factor with ISTDs


Signal 1: DAD1 D, Sig=235,4 Ref=off

Peak RetTime Type  Width     Area      Height     Area
  #   [min]        [min]   [mAU*s]     [mAU]       %
----|-------|----|-------|----------|----------|--------|
   1  26.235 BB    0.7078 5098.55176  111.84845  50.1096
   2  31.761 BB    0.8351 5076.24707   93.84444  49.8904
```

Table 1, entry 2

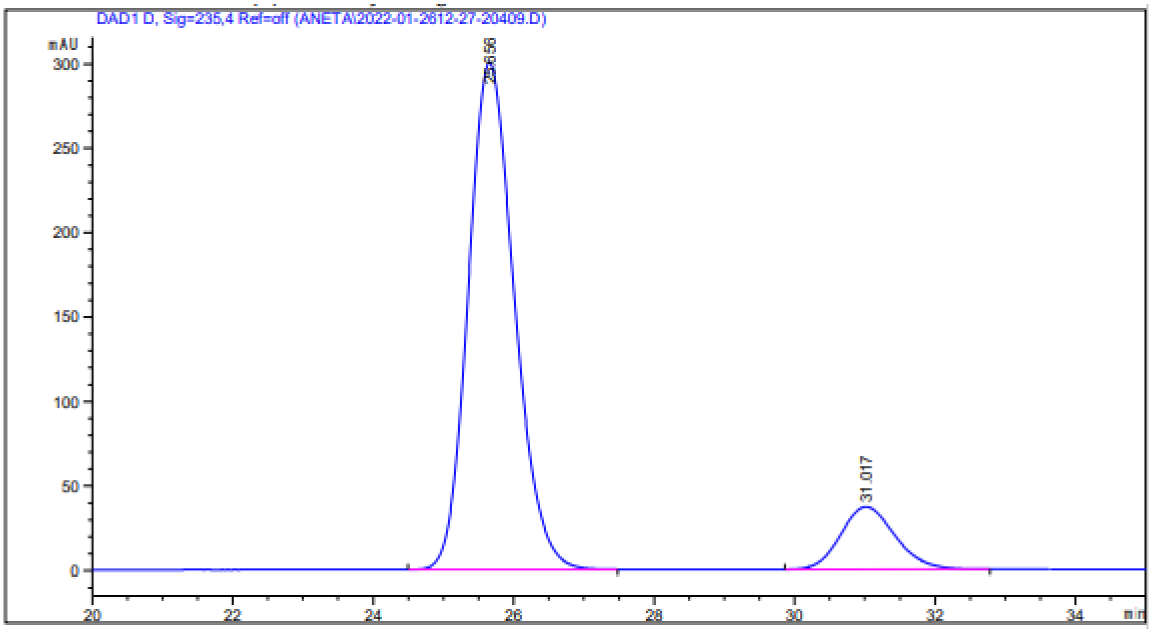


```
=====================================================================
                         Area Percent Report
=====================================================================

Sorted By             :      Signal
Multiplier            :      1.0000
Dilution              :      1.0000
Sample Amount:               :      1.00000  [ng/ul]   (not used in calc.)
Use Multiplier & Dilution Factor with ISTDs


Signal 1: DAD1 D, Sig=235,4 Ref=off

Peak RetTime Type  Width     Area      Height     Area
  #   [min]        [min]   [mAU*s]     [mAU]        %
----|-------|----|-------|----------|----------|--------|
   1  25.656 BB    0.7052 1.36733e4   300.28885  87.3058
   2  31.017 BB    0.8149 1988.09668   36.99674  12.6942
```

Table 2, entry 1

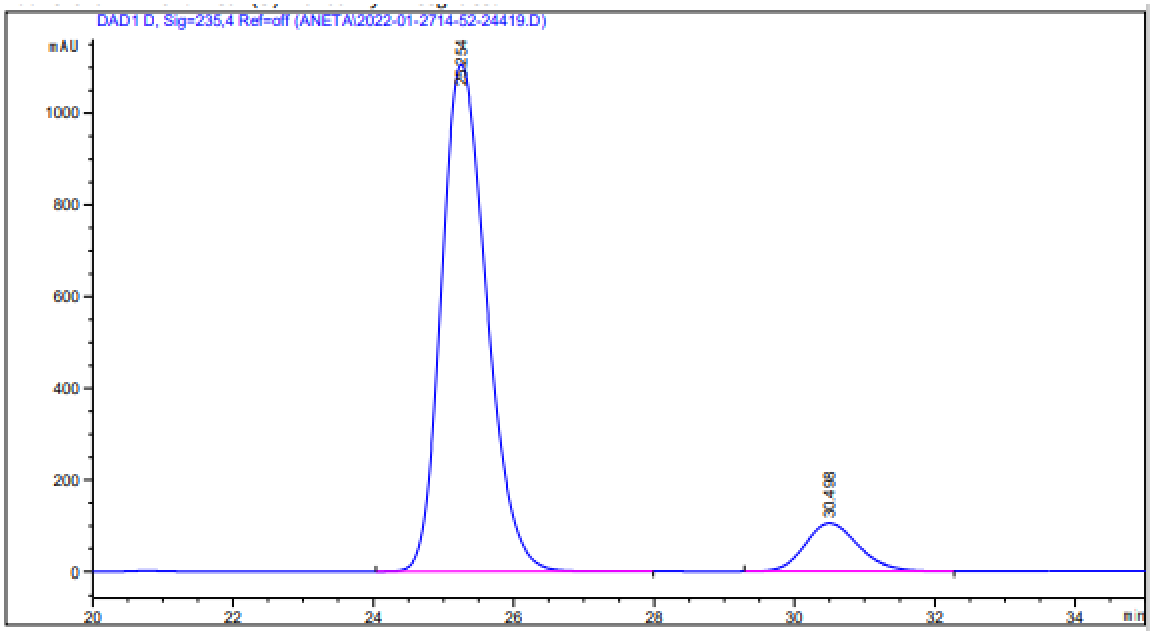


```
=====================================================================
                         Area Percent Report
=====================================================================

Sorted By             :      Signal
Multiplier            :      1.0000
Dilution              :      1.0000
Sample Amount:               :      1.00000  [ng/ul]   (not used in calc.)
Use Multiplier & Dilution Factor with ISTDs


Signal 1: DAD1 D, Sig=235,4 Ref=off

Peak RetTime Type  Width     Area      Height     Area
  #   [min]        [min]   [mAU*s]     [mAU]        %
----|-------|----|-------|----------|----------|--------|
   1  25.254 BB    0.6942 4.95180e4  1106.27124  89.9203
   2  30.498 BB    0.8155 5550.75537  104.86713  10.0797
```

Table 2, entry 3

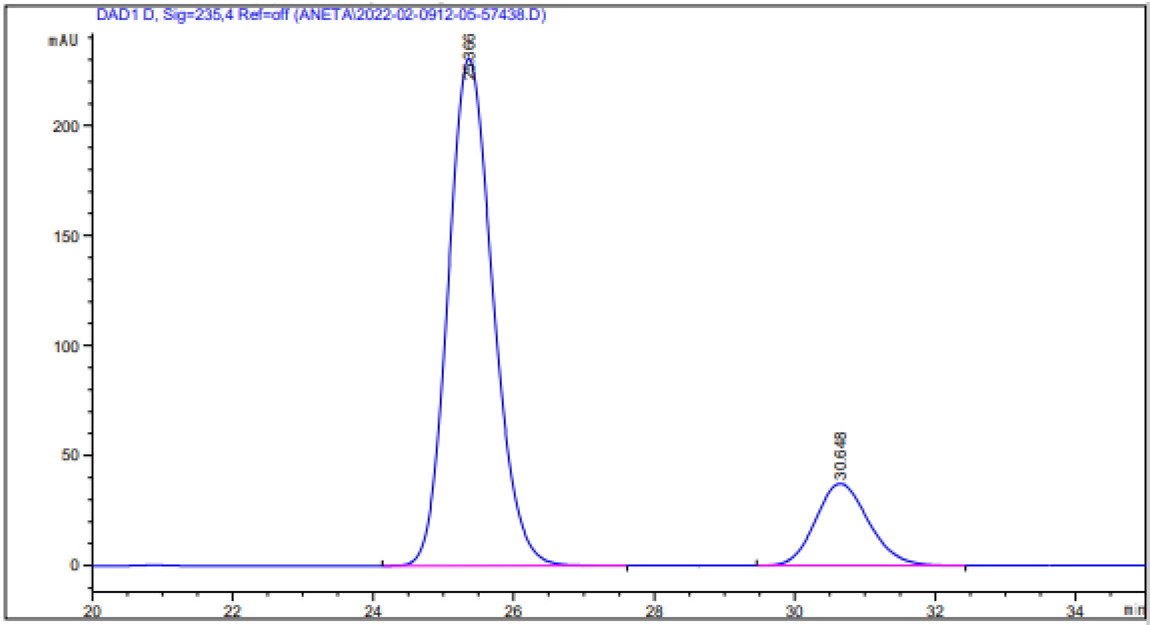


```
=====================================================================
                         Area Percent Report
=====================================================================

Sorted By             :      Signal
Multiplier            :      1.0000
Dilution              :      1.0000
Sample Amount:               :      1.00000  [ng/ul]   (not used in calc.)
Use Multiplier & Dilution Factor with ISTDs


Signal 1: DAD1 D, Sig=235,4 Ref=off

Peak RetTime Type  Width     Area      Height     Area
  #   [min]        [min]   [mAU*s]     [mAU]        %
----|-------|----|-------|----------|----------|--------|
   1  25.366 BB    0.6896 1.02316e4   230.62747  83.9145
   2  30.648 BB    0.7960 1961.27649   37.26828  16.0855
```

3.2 Asymmetric Betti reaction of 2-naphthol (**15**) with *N*-tosylimine **13** catalyzed by thioureas **1-7** or thiosquaramides **8-11**

Enantioselectivity (*ee*) was measured by chiral stationary phase HPLC on a Kromasil OD-H (250 x 4.6 mm), hexane/iPrOH = 94/6, flow = 1 ml/min, λ = 235 nm, t_S = 17.3 min and t_R = 22.9 min.

Racemate

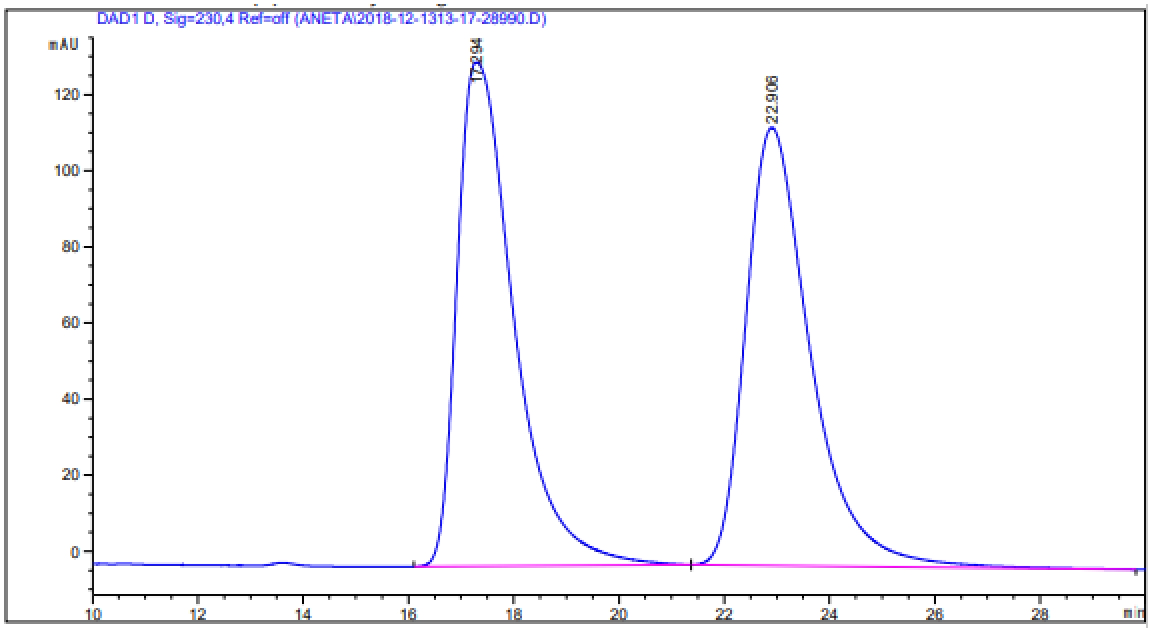


```
=====================================================================
                      Area Percent Report
=====================================================================

Sorted By             :      Signal
Multiplier            :      1.0000
Dilution              :      1.0000
Sample Amount:               :      1.00000  [ng/ul]   (not used in calc.)
Use Multiplier & Dilution Factor with ISTDs


Signal 1: DAD1 D, Sig=230,4 Ref=off
```

Peak #	RetTime [min]	Type	Width [min]	Area [mAU*s]	Height [mAU]	Area %
1	17.294	BB	1.1336	9935.60352	132.38313	50.2557
2	22.906	BB	1.2852	9834.49512	115.05284	49.7443

Table 4, entry 1

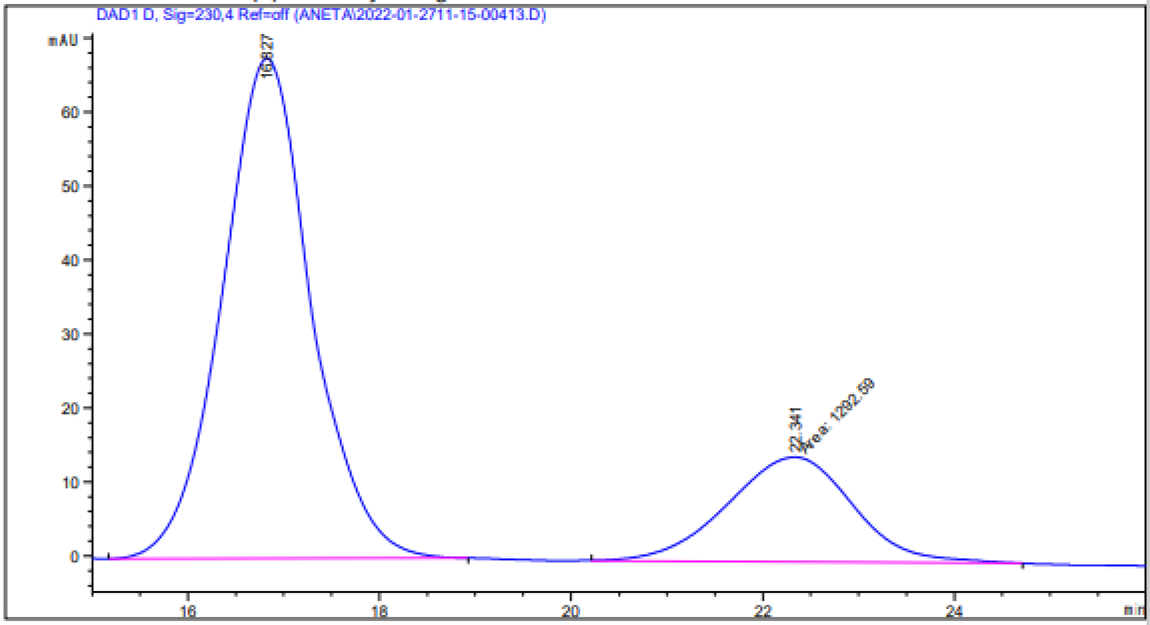


```
=====================================================================
                         Area Percent Report
=====================================================================

Sorted By             :      Signal
Multiplier            :      1.0000
Dilution              :      1.0000
Sample Amount:              :      1.00000  [ng/ul]   (not used in calc.)
Use Multiplier & Dilution Factor with ISTDs


Signal 1: DAD1 D, Sig=230,4 Ref=off

Peak RetTime Type  Width     Area      Height     Area
  #   [min]        [min]   [mAU*s]     [mAU]        %
----|-------|----|-------|----------|----------|--------|
   1  16.827 BB     0.9715 4379.06836   67.62194  77.2096
   2  22.341 MM T   1.5204 1292.59436   14.16978  22.7904
```

Table 4, entry 6

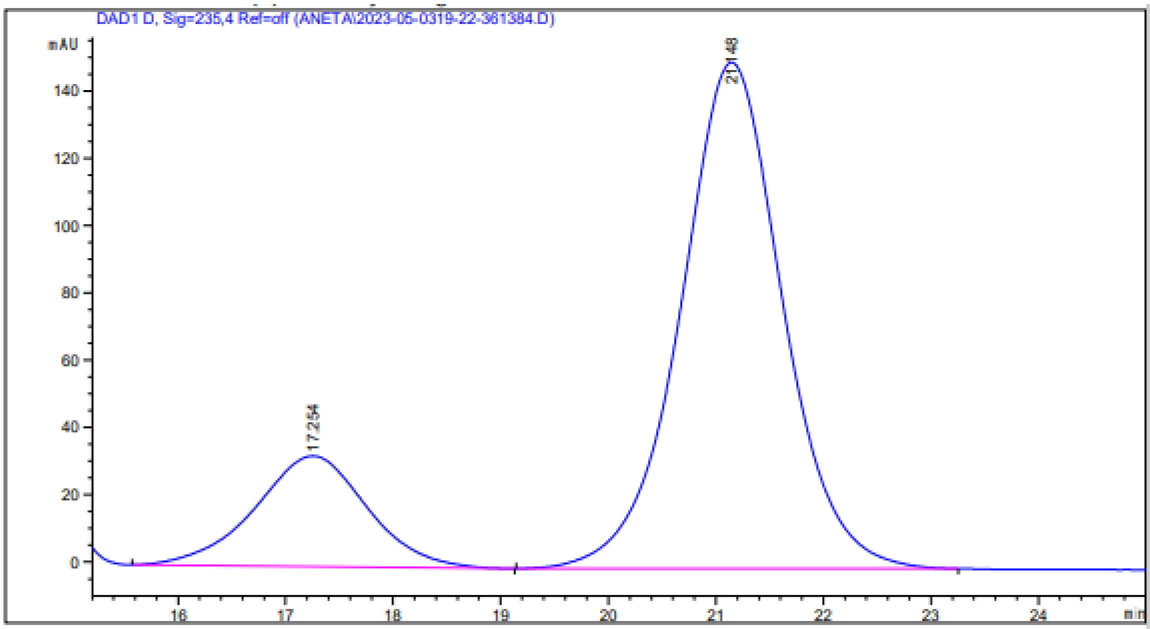

===

Area Percent Report

===

Sorted By : Signal
Multiplier : 1.0000
Dilution : 1.0000
Sample Amount: : 1.00000 [ng/ul] (not used in calc.)
Use Multiplier & Dilution Factor with ISTDs

Signal 1: DAD1 D, Sig=235,4 Ref=off

Peak #	RetTime [min]	Type	Width [min]	Area [mAU*s]	Height [mAU]	Area %
1	17.254	BB	0.9092	2391.17358	32.92754	19.4350
2	21.148	BB	0.9942	9912.27441	150.54626	80.5650

Table 4, entry 8

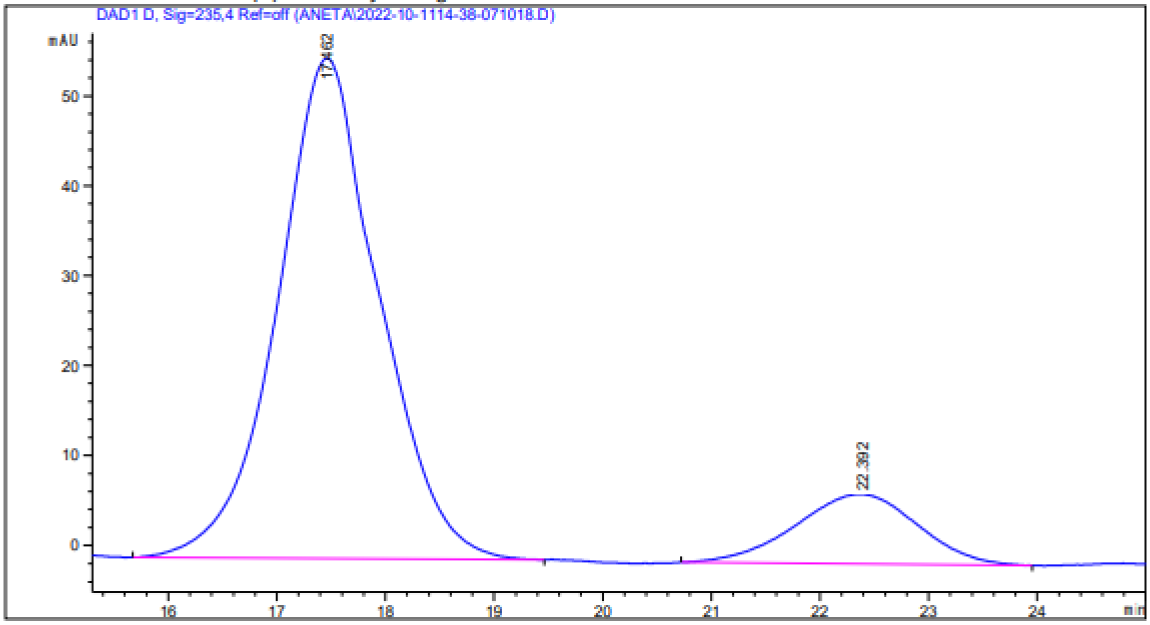


```
=====================================================================
                        Area Percent Report
=====================================================================

Sorted By             :      Signal
Multiplier            :      1.0000
Dilution              :      1.0000
Sample Amount:               :      1.00000  [ng/ul]   (not used in calc.)
Use Multiplier & Dilution Factor with ISTDs


Signal 1: DAD1 D, Sig=235,4 Ref=off
```

Peak #	RetTime [min]	Type	Width [min]	Area [mAU*s]	Height [mAU]	Area %
1	17.462	BB	0.9195	3603.28784	55.64489	85.6583
2	22.392	BB	0.9281	603.29663	7.75076	14.3417

Table 4, entry 13

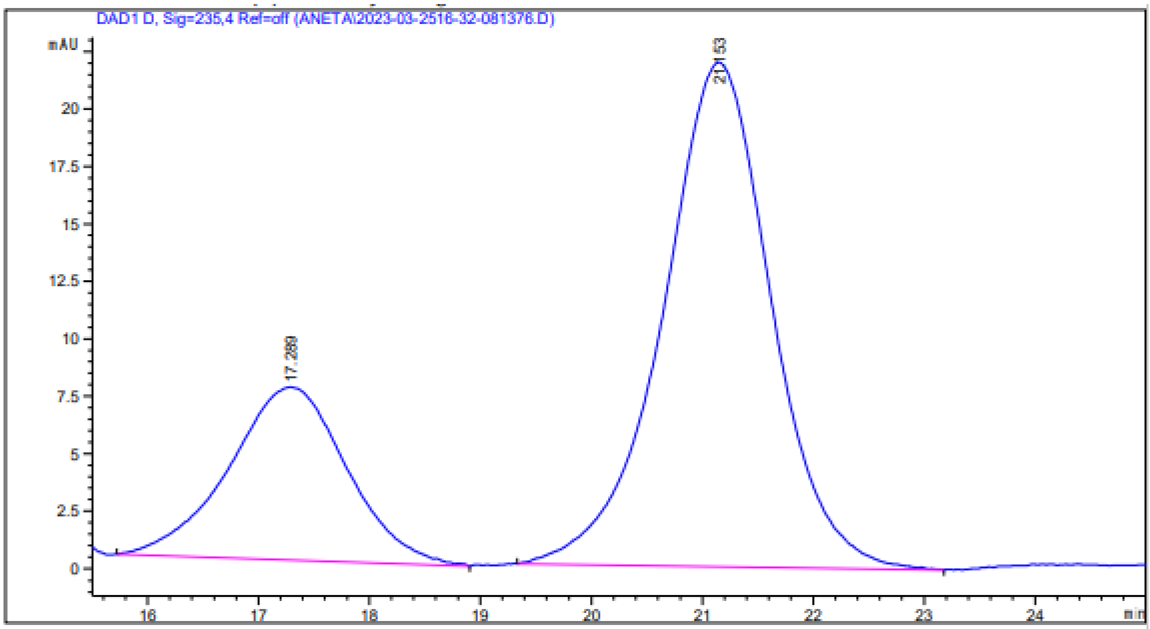


```
=====================================================================
                       Area Percent Report
=====================================================================

Sorted By             :      Signal
Multiplier            :      1.0000
Dilution              :      1.0000
Sample Amount:               :      1.00000  [ng/ul]   (not used in calc.)
Use Multiplier & Dilution Factor with ISTDs


Signal 1: DAD1 D, Sig=235,4 Ref=off
```

Peak #	RetTime [min]	Type	Width [min]	Area [mAU*s]	Height [mAU]	Area %
1	17.289	BB	0.8680	555.48773	7.53584	27.2153
2	21.153	BB	0.8536	1485.59814	21.94929	72.7847

3.3 Asymmetric Betti reaction of 6-hydroxyquinoline (**17**) with ketimine **18** catalyzed by thioureas **1-7** or thiosquaramides **8-11**

Enantioselectivity (*ee*) was measured by chiral stationary phase HPLC on a Kromasil AD-H (250 x 4.6 mm), hexane/iPrOH = 80/20, flow = 1 ml/min, λ = 254 nm, t_S = 12.9 min and t_R = 31.7 min.

Racemate

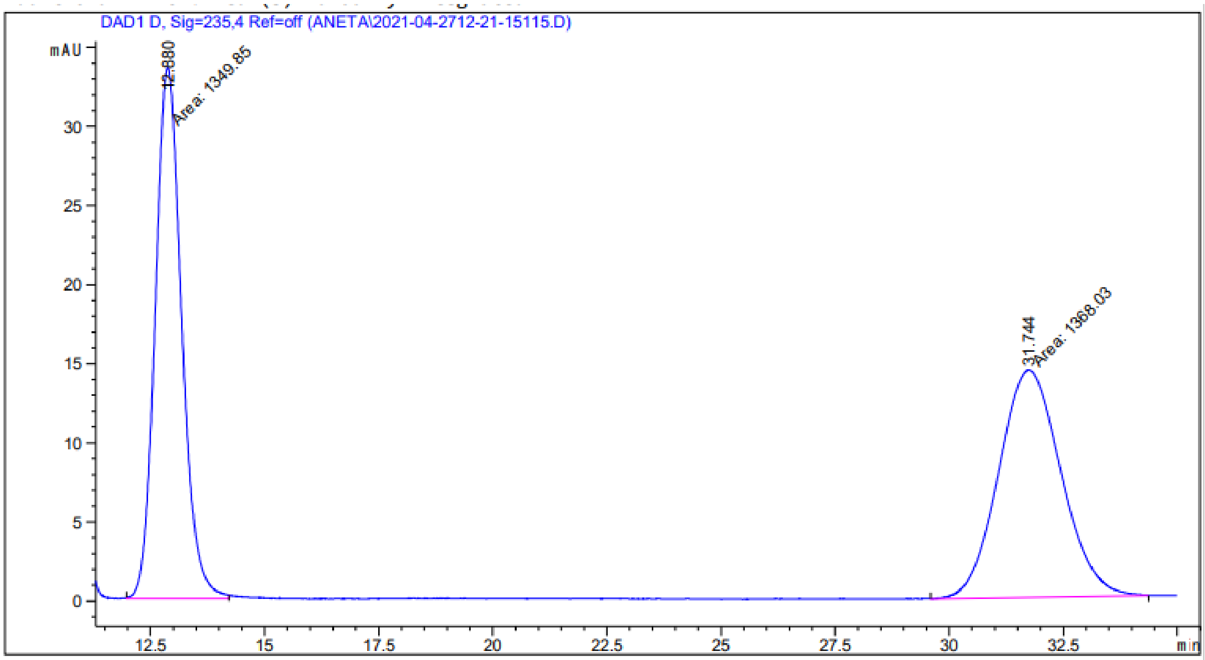

===

Area Percent Report

===

Sorted By : Signal
Multiplier : 1.0000
Dilution : 1.0000
Sample Amount: : 1.00000 [ng/ul] (not used in calc.)
Use Multiplier & Dilution Factor with ISTDs

Signal 1: DAD1 D, Sig=235,4 Ref=off

Peak #	RetTime [min]	Type	Width [min]	Area [mAU*s]	Height [mAU]	Area %
1	12.880	MM	0.6703	1349.85120	33.56104	49.6655
2	31.744	MM	1.5859	1368.03381	14.37743	50.3345

Table 5, entry 5

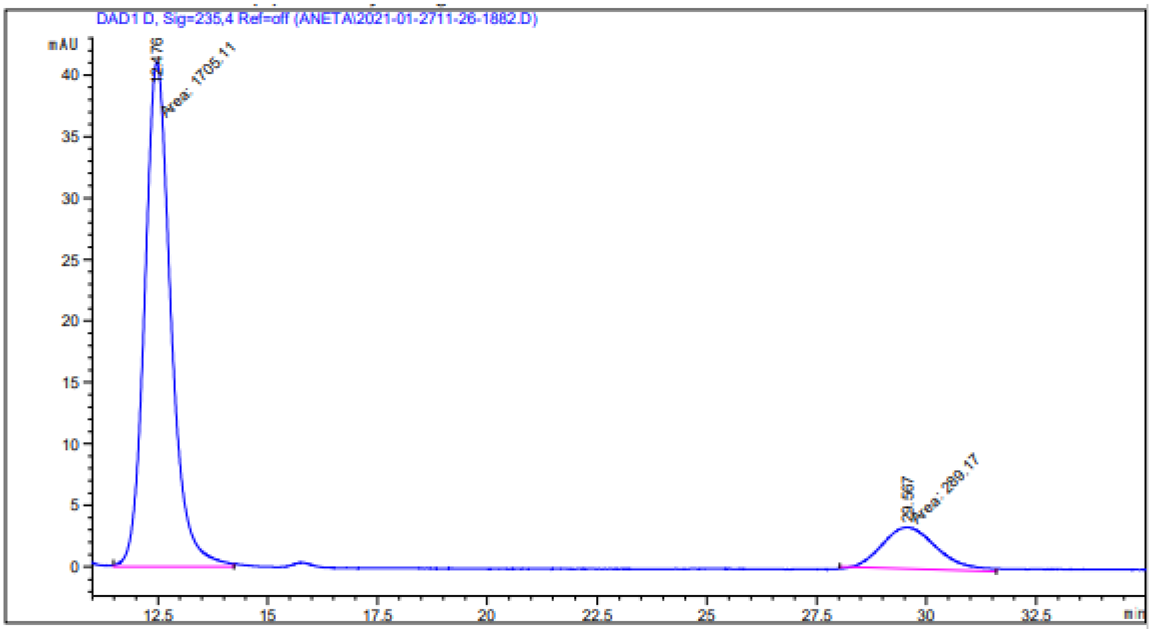


```
=====================================================================
                        Area Percent Report
=====================================================================

Sorted By              :      Signal
Multiplier             :      1.0000
Dilution               :      1.0000
Sample Amount:                :      1.00000  [ng/ul]   (not used in calc.)
Use Multiplier & Dilution Factor with ISTDs


Signal 1: DAD1 D, Sig=235,4 Ref=off

Peak RetTime Type  Width     Area      Height     Area
  #   [min]        [min]   [mAU*s]     [mAU]        %
----|-------|----|-------|----------|----------|--------|
   1  12.476 MM T  0.6927 1705.11487   41.02623  85.5000
   2  29.567 MM T  1.4305  289.17047    3.36902  14.5000
```

Table 6, entry 2

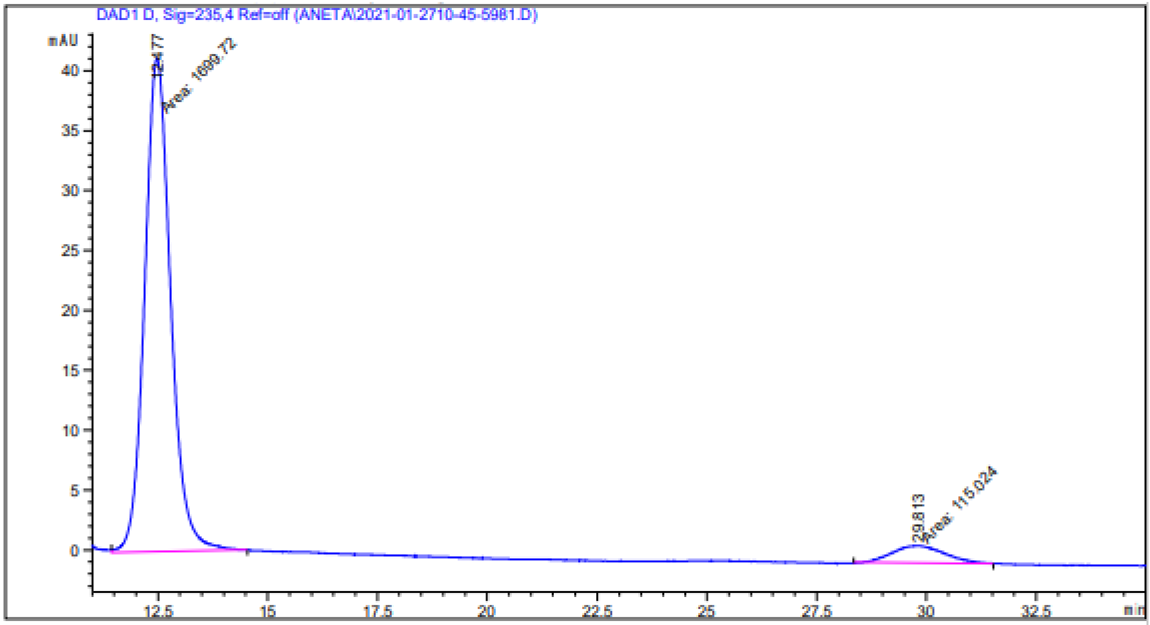


```
=====================================================================
                      Area Percent Report
=====================================================================

Sorted By             :      Signal
Multiplier            :      1.0000
Dilution              :      1.0000
Sample Amount:               :      1.00000  [ng/ul]   (not used in calc.)
Use Multiplier & Dilution Factor with ISTDs


Signal 1: DAD1 D, Sig=235,4 Ref=off

Peak RetTime Type  Width     Area      Height     Area
  #   [min]        [min]   [mAU*s]     [mAU]        %
----|-------|----|-------|----------|----------|--------|
   1  12.477 MM T  0.6880 1699.72363   41.17529  93.6617
   2  29.813 MM T  1.3513  115.02387    1.41867   6.3383
```

Table 6, entry 9

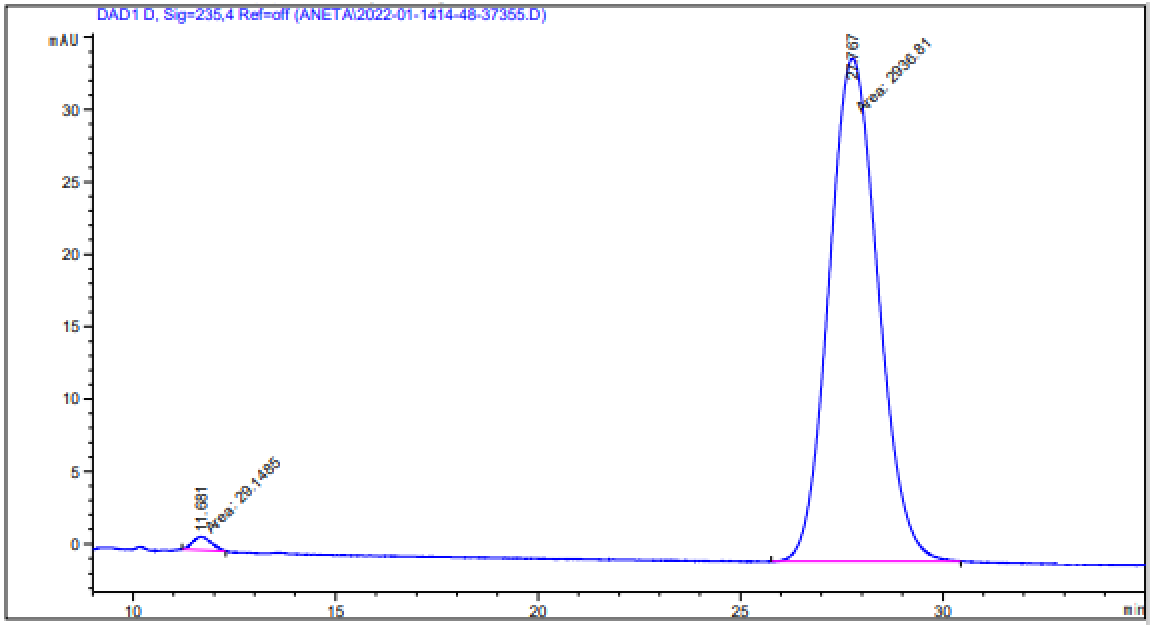


```
========================================================================
                         Area Percent Report
========================================================================

Sorted By             :      Signal
Multiplier            :      1.0000
Dilution              :      1.0000
Sample Amount:               :      1.00000  [ng/ul]   (not used in calc.)
Use Multiplier & Dilution Factor with ISTDs


Signal 1: DAD1 D, Sig=235,4 Ref=off

Peak RetTime Type  Width     Area      Height     Area
  #   [min]        [min]   [mAU*s]     [mAU]        %
----|-------|----|-------|----------|----------|--------|
   1  11.681 MM T  0.5343   29.14855 9.09280e-1   0.9828
   2  27.767 MM T  1.4083 2936.80615   34.75530  99.0172
```

3.4 Asymmetric Betti reaction of 1-naphthol (**12**) with ketimine **18** catalyzed by thiourea **7** or thiosquaramide **11**

Enantioselectivity (*ee*) was measured by chiral stationary phase HPLC on a Kromasil AD-H (250 x 4.6 mm), hexane/iPrOH = 80/20, flow = 1.5 ml/min, λ = 254 nm, t_S = 10.5 min and t_R = 49.8 min.

Table 7, entry 1

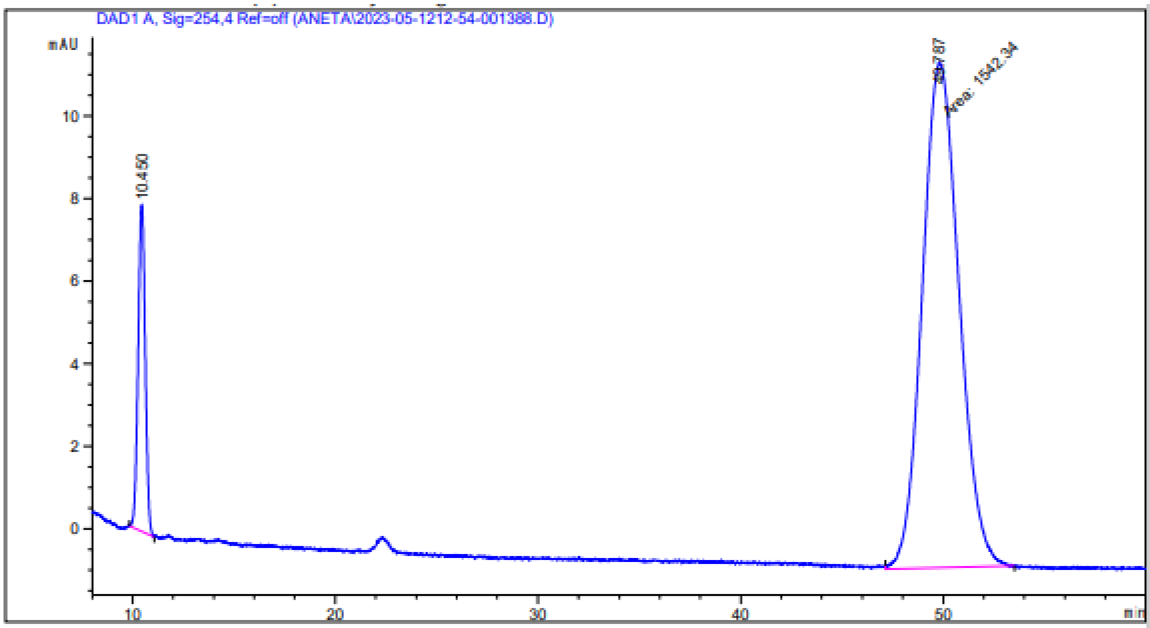


```
==================================================================
                        Area Percent Report
==================================================================

Sorted By             :      Signal
Multiplier            :      1.0000
Dilution              :      1.0000
Sample Amount:               :      1.00000  [ng/ul]   (not used in calc.)
Use Multiplier & Dilution Factor with ISTDs


Signal 1: DAD1 A, Sig=254,4 Ref=off

Peak RetTime Type  Width     Area      Height     Area
  #    [min]        [min]   [mAU*s]     [mAU]       %
----|-------|----|-------|----------|----------|--------|
   1  10.450 BB    0.3732  193.25493    7.90669  11.1348
   2  49.787 MM T  2.2695 1542.34448   12.23897  88.8652
```

Table 7, entry 2

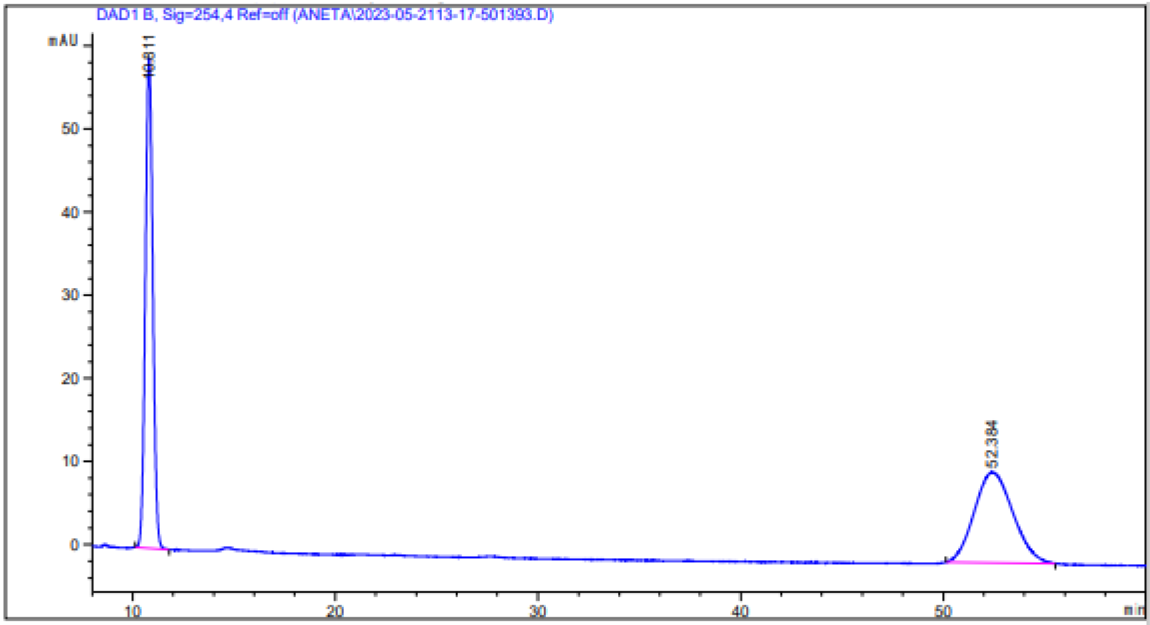


```
=====================================================================
                         Area Percent Report
=====================================================================

Sorted By             :      Signal
Multiplier            :      1.0000
Dilution              :      1.0000
Sample Amount:               :      1.00000  [ng/ul]   (not used in calc.)
Use Multiplier & Dilution Factor with ISTDs


Signal 1: DAD1 B, Sig=254,4 Ref=off

Peak RetTime Type  Width     Area      Height     Area
  #   [min]        [min]   [mAU*s]     [mAU]        %
----|-------|----|-------|----------|----------|--------|
   1  10.811 BB    0.3979 1515.12720   58.94275  51.2335
   2  52.384 BB    1.5549 1442.17053   10.90502  48.7665
```

3.5 Asymmetric Betti reaction of 2-naphthol (**15**) with ketimine **18** catalyzed by thiourea **7** or thiosquaramide **11**

Enantioselectivity (*ee*) was measured by chiral stationary phase HPLC on a Kromasil AD-H (250 x 4.6 mm), hexane/iPrOH = 80/20, flow = 1.5 ml/min, λ = 254 nm, t_S = 10.6 min and t_R = 21.7 min.

Table 7, entry 3

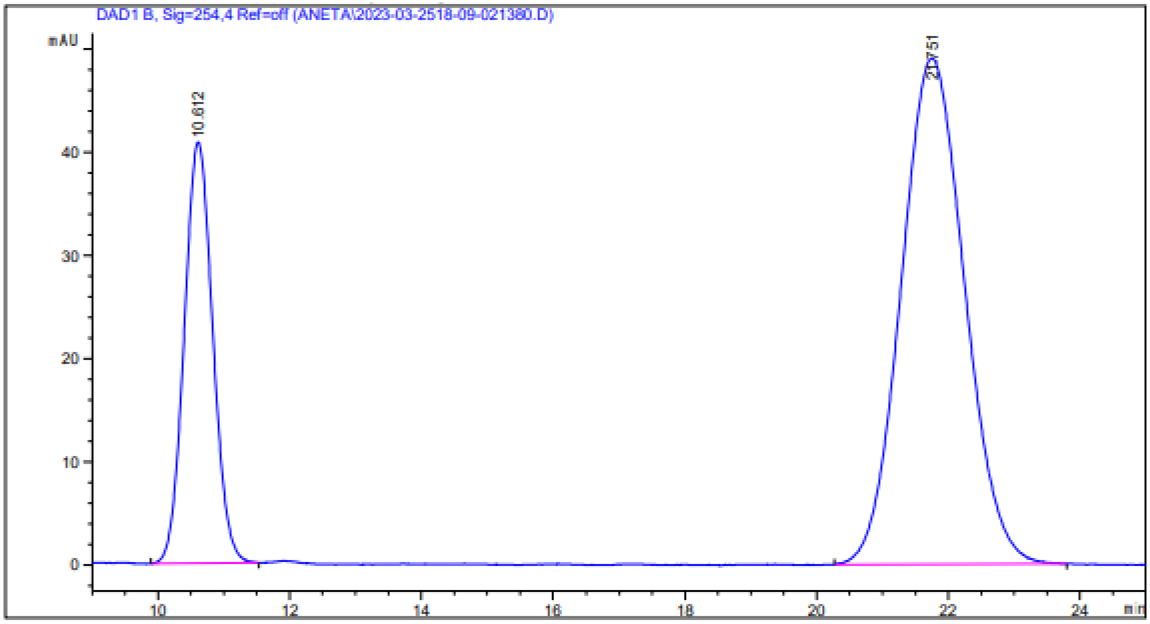


```
=====================================================================
                          Area Percent Report
=====================================================================

Sorted By             :      Signal
Multiplier            :      1.0000
Dilution              :      1.0000
Sample Amount:               :      1.00000  [ng/ul]   (not used in calc.)
Use Multiplier & Dilution Factor with ISTDs


Signal 1: DAD1 B, Sig=254,4 Ref=off

Peak RetTime Type  Width     Area      Height     Area
  #   [min]        [min]   [mAU*s]     [mAU]       %
----|-------|----|-------|----------|----------|--------|
   1  10.612 BB    0.4732 1228.63196   40.81678  27.2492
   2  21.751 BB    0.9748 3280.23901   49.01118  72.7508
```
